# Exploring the impact of information and communication technologies on loneliness and social isolation in community-dwelling older adults: a scoping review of reviews

**DOI:** 10.1186/s12877-024-04837-1

**Published:** 2024-03-02

**Authors:** Mari Gunnes, Ida-Camilla Løe, Jorid Kalseth

**Affiliations:** 1 Department of Health Research, SINTEF Digital, Trondheim, Norway; 2 Department of Technology Management, SINTEF Digital, Steinkjer, Norway

**Keywords:** Social isolation, Loneliness, Information and communication technologies, Older adults, Scoping review of reviews, Knowledge synthesis

## Abstract

**Background:**

Recognizing the escalating public health concerns of loneliness and social isolation in aging populations, this study seeks to comprehensively explore the potential of information and communication technology (ICT)-based interventions to address these issues among older adults. This scoping review of reviews aims to map and synthesize existing evidence on the effectiveness and scope of ICT interventions targeting loneliness and social isolation in community-dwelling older adults, elucidating types of technology, impacts, facilitators, barriers, and research gaps.

**Methods:**

Following the Joanna Briggs Institute framework, we systematically searched eight diverse databases identifying relevant published reviews. We included English-written, peer-reviewed reviews of all types, with no limits regarding time of publication about ICTs targeting loneliness and/or social isolation for community-dwelling older adults. Eligible reviews were analysed and summarized, offering a holistic narrative of the reported types of ICTs and their impact, the identified facilitators and barriers influencing the implementation and adoption of ICT interventions, and the research gaps identified in the literature.

**Results:**

The review included 39 publications published between 2012 and 2024, spanning systematic, scoping, and reviews of reviews. Various ICTs were reported, primarily social media virtual communities, followed by video-mediated friendly visits, conversational agents, social robots, exergames and online gameplay. Predominantly positive impacts on mitigating social isolation and loneliness were evident for these ICTs, although methodological diversity and contradictory findings complicated definite conclusions. Facilitators and barriers encompassed individual competencies, access and usage, and intervention design and implementation. Research gaps involved targeting specific subgroups, exploring innovative technologies, incorporating diverse study designs, improving research methodologies, and addressing usability and accessibility. Future research should focus on identifying elderly individuals who can benefit the most from ICT use, exploring novel technologies, using a wider range of study designs, and enhancing usability and accessibility considerations.

**Conclusions:**

This review sheds light on the diverse range of ICTs, their impact, and the facilitators and barriers associated with their use. Future investigations should prioritize refining outcome measures, addressing gender differences, and enhancing the usability and accessibility of interventions. The involvement of older adults in the design process and the exploration of technological training interventions hold promise in overcoming barriers.

**Supplementary Information:**

The online version contains supplementary material available at 10.1186/s12877-024-04837-1.

## Introduction

Loneliness and social isolation are increasingly being recognized as public health concerns in our aging society and were addressed as a priority issue as part of the Decade of Healthy Aging by the World Health Organization (WHO) [1]. Among elderly individuals, loneliness and social isolation constitute a significant burden both to the individual and their families and to the health care system and society at large. On the individual level, it has a negative impact on older adults’ physical, mental and emotional health and well-being, as it may increase the risk of chronic health conditions, such as cardiovascular disease, stroke, dementia and depression [2–6]. Concurrently, the burden for caregivers [7] and the health care system are increasing as healthcare costs are expanding [8].

Loneliness and social isolation are complex and multifaceted concepts that may be defined in various ways, as they are distinct but related concepts. In general, loneliness can be described as the subjective feeling of being alone or isolated, while social isolation may be characterized as the objective state of having few social relationships or infrequent social contact with others [6]. More specifically, loneliness is described as the subjective painful feeling of the absence of a social network or companion or the perception of unmet emotional and social needs resulting from a mismatch between the desired and actual experience of the quality or quantity of social relationships. Social isolation, however, is understood as the objective state of a lack of interactions with others and the wider community or a lack of social relationships [1]. Factors contributing to loneliness and social isolation in older adults encompass a range of predictors, including but not limited to, living alone, experiencing a lack of social support resulting from the loss of a significant other, family separation, and having few friends. Additionally, being a caregiver for a spouse, and suffering health conditions such as depression, anxiety, dementia, serious illness, decreased mobility, and loss of independence can also play significant roles in fostering feelings of loneliness and social isolation [9–11].

Although there is no global estimate of the proportion of elderly individuals who experience loneliness and social isolation in their communities, the prevalence is assumed to range from 5 to 50 %, depending on the country and population being studied, the design of the studies, and the definition of the concepts [12, 13]. For instance, in European countries and within the age range of 60 and 80 years, the prevalence of frequently feeling lonely is shown to be between 5 and 10 %. The prevalence dramatically increases in advanced age (i.e., 80+), with studies stating that between 40 and 50 % report often feeling lonely [12, 13]. Other studies indicated a prevalence of loneliness ranging from 25 to 29 % in the USA [14], 25 to 32 % in Latin America [15], 18 to 44 % in India [15, 16], and 3.8 to 29.6 % in China [15, 17]. A limited number of studies have estimated the prevalence of social isolation; however, there are a few indicating rates of 24 % in the USA [18], 10 to 43 % in North America [19], and 20 % in India [20]. As the global population aged 60 years and older is estimated to increase from 12 to 22 % between 2015 and 2050 [21], the number of older adults who are living alone or who are isolated from others is increasing.

Several interventions and strategies have exhibited promise in reducing social isolation and loneliness, primarily focusing on individual and relationship-level interventions, with sparse evidence for interventions at the community or societal level [11, 22, 23]. The efficacy of interventions and strategies in reducing social isolation and loneliness in older adults, as well as determining the optimal candidates for these interventions, remains unclear [1, 9]. Recently, the role of technology has been increasingly important, with digital interventions garnering particular interest due to the rise in the use of technology over the past decade [1]. Attendant physical distancing measures during the COVID-19 pandemic have also increased the salience of these topics [24–26]. Information and communication technologies (ICTs), which refers to a broad range of technologies that provide access to information through telecommunications, includes digital and networked technologies, such as messaging services, online discussion groups, social network sites, and virtual artificial intelligence companions [27]. ICT interventions have the potential to provide socialization and connection opportunities, thus mitigating social isolation and loneliness in older adults. However, the range and extent to which ICTs are effective in reducing the risk of social isolation and loneliness in the elderly are not yet fully established [1, 28].

A scoping review is a type of systematic review that aims to map the extent, range, and characteristics of research on a particular topic [29, 30]. Given the rapid growth of research in the field of ICTs addressing loneliness and social isolation, primary reviews are emerging. Loneliness and social isolation in older adults are complex issues influenced by various factors. To provide a holistic perspective by examining different dimensions of ICTs, a scoping review of reviews was considered the most suitable approach to explore and summarize the emerging evidence in this research field. By synthesizing and summarizing the current evidence, this review aims to provide an overview of available reviews in the field and identify areas for future research. Notably, our emphasis on exploring barriers, facilitators and knowledge gaps in addressing loneliness and social isolation among older adults, distinguishes our review, aimed to offer valuable insights for future research endeavours to enhance the impact of such ICT interventions on loneliness and social isolation among older adults.

### Scoping review objectives

In this scoping review of reviews, the overall aim was to broadly map and synthesize the existing evidence from published reviews on ICT interventions targeting loneliness and social isolation among community-dwelling older adults to identify the (i) types of ICTs, (ii) their impact, (iii) the facilitators and barriers associated with their use, and (iv) the research gaps in this field.

## Methods

This scoping review of reviews was conducted in line with the framework described and outlined by the Joanna Briggs Institute (JBI) [29], based on the previous work of Arksey and O’Malley [31] and Levac and colleagues [32]. The JBI approach to conducting and reporting scoping reviews is congruent with The Preferred Reporting Items for Systematic Reviews and Meta-Analyses (PRISMA) Extension for Scoping Reviews checklist [33], which was used as a guide for reporting the results. The process followed the five key stages: 1) identifying the research question, 2) identifying relevant studies, 3) selecting studies, 4) charting the data, and 5) collating, summarizing and reporting the results. The sixth, and nonmandatory stage, 6) consultation, was not conducted due to practical constraints, including limited resources within the study timeframe and the unavailability of stakeholders.

### Stage 1: identifying the research questions

First, we conducted a preliminary literature search with the aim of obtaining a certain overview of concepts, terminology, relevant keywords and research literature in the field. After the initial search, the following research questions were developed and included: 1) What types of ICT interventions have been reviewed and evaluated for their impact on mitigating loneliness and social isolation among community-dwelling older adults? 2) What is the indicated evidence from reviews regarding the effectiveness of ICT interventions in reducing loneliness and social isolation among older adults? 3) What are the identified facilitators and barriers that influence the implementation and adoption of ICT interventions? 4) What are the gaps identified in the literature on this field?

### Stage 2: identifying relevant studies

Eligibility criteria were set (Table 1), and search terms were identified using the population-concept-context (PCC) framework provided by JBI [29]. Keywords for the population included elderly, old adults, aged, aging and senior. Keywords pertaining to the concept included digital technology, ICT interventions, e-interventions, Internet and social media, and those for the outcome included loneliness, social isolation and social participation. We used Boolean phrases, combinations of keywords, and filters for reviews where applicable. The full search strategy can be found in Appendix 1.

**Table 1:** Eligibility criteria

**PCC element**	**Eligibility criteria**
**Population**	**Inclusion:** Reviews encompassing older adults without defining a specific age threshold. **Exclusion:** Reviews exclusively centred on populations with specific diseases or illnesses (e.g., older adults with major neurological disorders), or solely focused on informal and formal caregivers in the context of older adults.
**Concept**	**Inclusion:** Interventions utilizing information and communication technology (ICT) with the aim of mitigating loneliness and/or social isolation. **Exclusion:** Interventions utilizing telemedicine or welfare technology lie beyond the scope of this review.
**Context**	**Inclusion:** Older adults living in community settings (i.e., those living in their own homes, as well as those in combination with nursing homes or residential care). **Exclusion:** Reviews focused solely on hospital settings or exclusive nursing home settings are outside the scope of consideration.
**Types of sources of evidence**	**Inclusion:** Peer-reviewed reviews of all types. No restriction on the publication year but limited to the English language. **Exclusion:** Nonreviews (e.g., original research papers, editorials, opinion pieces/commentary, book chapters, conference proceedings, protocols, reports, preprints).

We included literature reviews in English about ICTs targeting loneliness and/or social isolation for community-dwelling older adults. Peer-reviewed reviews of all types (including outcome assessments based on qualitative, quantitative, or mixed methods) published in academic journals were eligible. Studies were excluded if they solely focused on subpopulations of older adults (e.g., those living in nursing homes or hospital settings with a specific disease or illness). No limits regarding year of publication were applied.

Eight databases (i.e., PubMed, Scopus, Medline, Cochrane Library, Web of Science, PsychInfo, CINAHL, and Epistemonikos) were systematically searched by two researchers for relevant literature from their inception until the dates that the search was conducted (16th to 18th August 2022). The databases were selected to be comprehensive and to cover a broad range of disciplines. To fill potential gaps in the initial literature search and to provide a more complete picture of the literature on the topic of interest, we conducted an additional search for literature in May 2023. First, we conducted backwards citation chaining (6th of May 2023), which involved manually searching the reference lists of the included reviews. Second, we conducted a forward citation chaining (22 to 23 May), which involved examining the studies that had cited the identified reviews. Furthermore, the initial search was updated by systematically searching the same databases (i.e., all but PsychInfo due to lack of institutional access) from the time of the initial search in mid-August 2022 until the most recent search conducted on February 13th, 2024. Backwards and forward citation chaining were once again conducted on February 15th, 2024, following the same procedure described above.

### Stage 3: selecting studies

Citations from each database were captured in Zotero reference manager and exported to Rayyan. The latter is a free web-based software platform designed to help researchers collaborate in producing systematic reviews and other knowledge synthesis projects. After excluding duplicates in Rayyan, two researchers independently screened the titles and abstracts to exclude irrelevant studies and to identify reviews for possible inclusion. Reviews selected for possible inclusion were exported to Excel and examined and confirmed for selection based on the eligibility criteria. Disparities were resolved through discussion between two of the authors.

### Stage 4: charting the data

Reviews that met the inclusion criteria were reviewed and extracted independently by the authors. The following information was extracted into an Excel spreadsheet: general characteristics of the review (i.e., authors, year of publication, title, journal), type of review (e.g., scoping, systematic, qualitative), number and study design of the included primary studies, population studied (including age range and gender, when applicable), type of intervention/technology reported, outcome measurements, findings/results, implications for practice (if applicable), author’s conclusions, and author’s suggestion for future research.

Once the data extraction was completed, the results were organized into broader subtopics that emerged from the included reviews. The following topics arose: (i) types of ICT interventions described; (ii) outcome assessments of loneliness and/or social isolation, (iii) effect or impact of the ICT interventions on loneliness and/or social isolation, (iv) facilitators and barriers of using ICT interventions, and (v) identified research gaps for future research.

### Stage 5: collating, summarizing, and reporting the results

The included reviews were summarized and presented in evidence tables and illustrated with relevant figures. We examined the types of ICT, the reported effectiveness or impact of ICT interventions in reducing loneliness and social isolation among older adults, the identified facilitators and barriers that influenced the implementation and adoption of ICT interventions, and summarized the gaps identified in the literature on ICT intervention. This allowed us to create a narrative summary of the study objectives and findings.

## Results

### Literature search

**Fig. 1:**
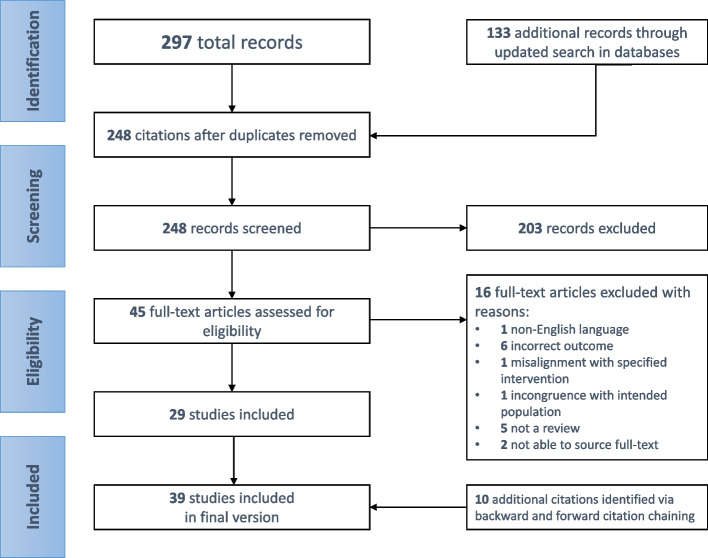
PRISMA flow chart illustrating the study selection process

The search and selection process for the review is summarized in Fig. 1. Our initial systematic search in medio August 2022 identified 297 citations, from which 168 were unique after removing duplicates. Upon conducting an updated search in mid-February 2024, we identified an additional 133 citations, with 80 being unique after duplicate removal. The eligibility of titles and abstracts was determined based on predefined criteria, resulting in 45 citations being considered relevant. Full texts were obtained and screened for inclusion, and 16 citations were excluded for various reasons, such as incorrect outcome (*n* = 6), not being a review (*n* = 5), inability to source full text (*n* = 2), non-English language (*n* = 1), incongruence with the intended population (n = 1), or misalignment with the specified intervention (n = 1). Backwards and forward citation chaining conducted in May 2023 and February 2024, respectively, led to the inclusion of four [34–37] and six [28, 38–42] additional relevant reviews, respectively, resulting in a total of 39 studies included in the present scoping review. Detailed characteristics of the included reviews are shown as a structured table and as a narrative summary in Appendix 2.

### Characteristics of the reviews

Heterogeneity in the included reviews was found in terms of the type of reviews, study designs within the primary sources, definition of older adults, level of evidence and outcome synthesis.

**Fig. 2:**
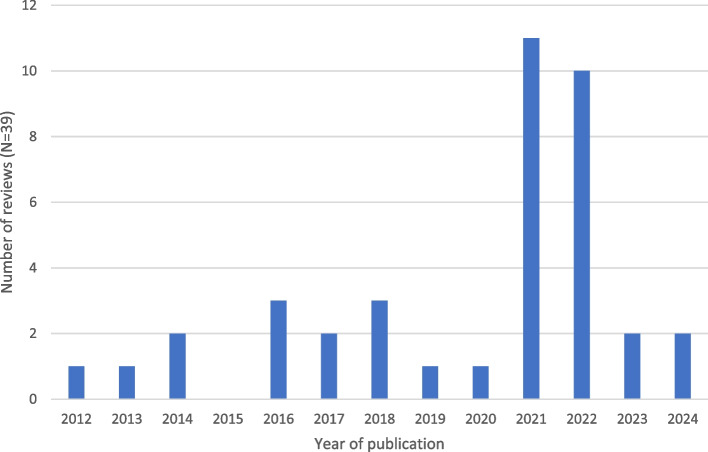
Number of reviews (*N* = 39) by year of publication

The included reviews were published from 2012 to 2024, with a higher frequency of publications in 2021 and 2022 (Fig. 2). Notably, there was a significant shift in the number of publications from 2020 (*n* = 1) to 2021 (*n* = 11).

**Fig. 3:**
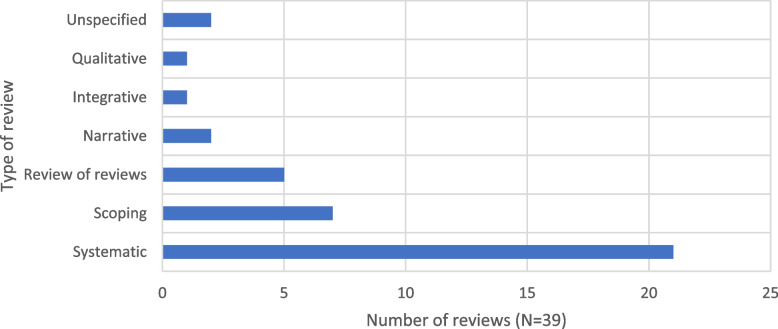
Number of reviews (*N* = 39) by type of review

Among the included papers, the most prevalent type of reviews obtained was systematic reviews (*n* = 21) [41, 43–62]. This was followed by scoping reviews (*n* = 7) [36, 40, 42, 63–66] and reviews of reviews (*n* = 5) [28, 34, 38, 39, 67]. Additionally, various types of reviews were identified, including narrative [37, 68], integrative [69], qualitative [70], and two unspecified reviews [35, 71] (Fig. 3).

Most reviews were conducted in the North American, European and Western Pacific regions, with several of the primary studies represented from the USA and the Netherlands. None of the reviews focused on research conducted in low- and middle-income countries as defined by the Organization for Economic Co-operation and Development (OECD) [72].

The definition of ‘older adults’ exhibited substantial variation across reviews and the primary studies they encompassed. One study included participants aged 45 and above [68], while several reviews incorporated primary sources with participants in their early 50 s and older [28, 35, 37–39, 47, 48, 51, 54, 55, 60, 62–67]. Other reviews set an age threshold of 60 years and above [34, 36, 40–42, 44–46, 49, 50, 52, 59, 61, 71, 73], and only two reviews exclusively focused on participants aged 70 and above [58, 70]. Moreover, two reviews encompassed studies involving participants across the lifespan, ranging from children to older adults [53, 56], although the focus of the results primarily centred around older adults, aligning with the scope of this review.

Regarding the distribution of gender among participants, most reviews did not provide gender-specific information. Among the reviews that reported the gender distribution, a general comment on ‘mixed’ was reported [35, 45, 67], while one review reported the number of females in each study [61]. Six reviews indicated a predominance of women included [36, 37, 40, 55, 58, 71], while one review reported a higher proportion of men included [47].

In terms of the setting or context of the studies, most reviews included a combination of elderly individuals living at home and those residing in nursing homes or residential care facilities. However, certain reviews focused exclusively on older adults living in their home environment [40, 47, 48, 53, 59, 62, 64]. This indicated that some reviews specifically targeted the general older adult population, while others encompassed studies involving individuals with multiple chronic conditions.

Some of the reviews explicitly focused on interventions to reduce social isolation [44, 45, 70, 73], while others explicitly focused on loneliness [36, 37, 41, 50, 58]. Most reviews, however, included papers with interventions addressing both social isolation and loneliness [28, 38, 40, 46, 48, 49, 51, 52, 62, 66, 67, 69]. Others focused on loneliness and/or social isolation alongside other health outcomes of interest, such as anxiety, depression, social participation, social interaction, social connectedness, and quality of life [34, 39, 42, 47, 53–57, 59, 61, 63, 64, 68, 71, 74].

A wide range of outcome measures and assessments were utilized across the different studies. Among these, the University of California Los Angeles (UCLA) Loneliness scale, including various modifications of the scale, emerged as the most frequently used measure, as referenced in several reviews. Following this, the De Jong Gierveld loneliness scale was also commonly employed. Several other outcome measures were mentioned, such as the Lubben Social Network Scale, Kamphuis’ loneliness scale, the Center for Epidemiological Studies Depression Scale, the Rosenberg Self-Esteem Scale. Notably, some reviews did not specify the outcome assessments utilized in the included studies, or they employed indirect measures or proxies for loneliness and social isolation [47, 48, 55–57, 61, 63]. In reviews that incorporated qualitative primary studies, either exclusively or in combination with quantitatively designed studies, the reported results were derived from analyses of interviews and observations conducted from various perspectives [36, 38, 40, 44, 49, 54, 61, 63, 68, 69, 74]. Different approaches, such as ethnographic studies, observational research, and individual or focus interviews, were utilized to obtain these results.

### Types and impact of ICT interventions

**Fig. 4:**
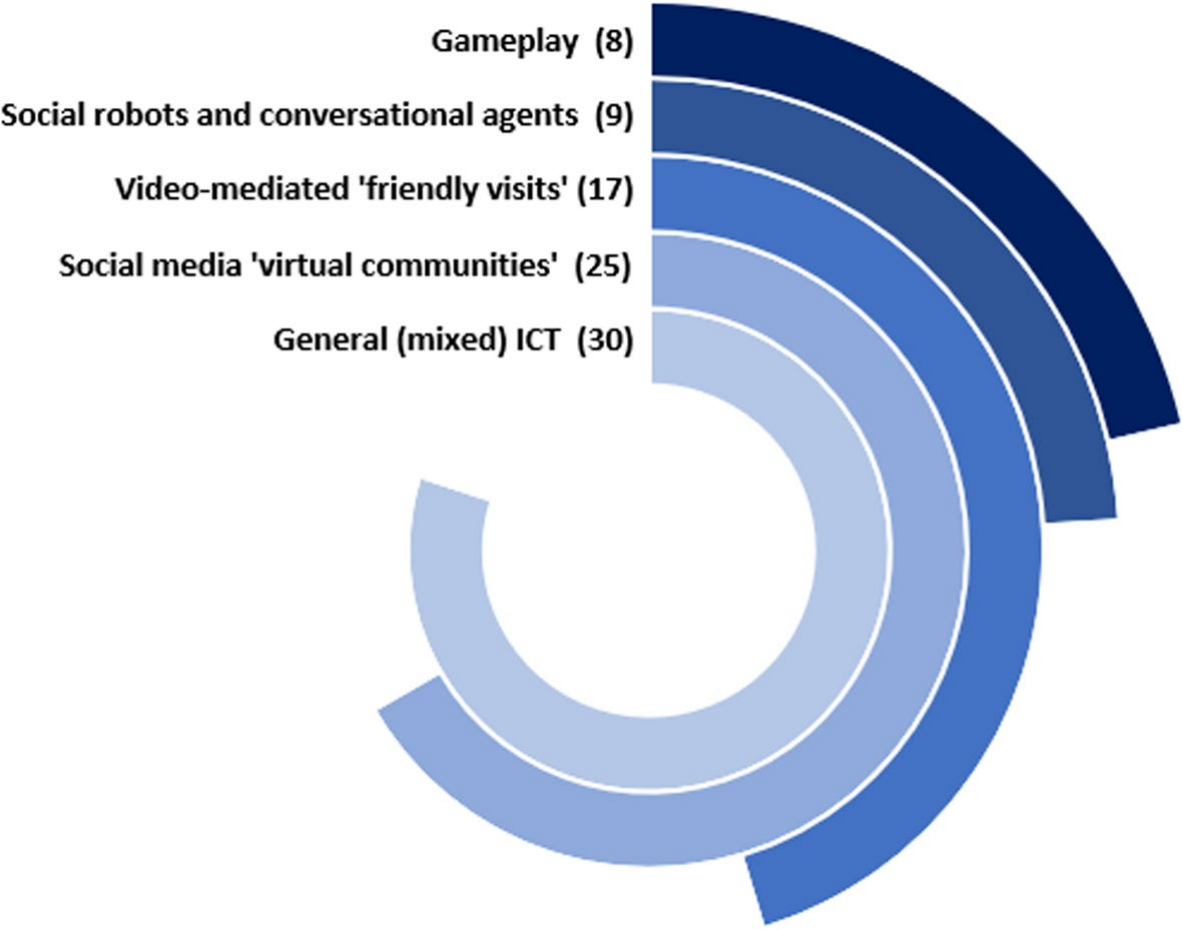
Distribution of ICT interventions in the included reviews (*N* = 39). The numbers written in parentheses indicate the number of studies included in each category. Note that multiple categories were covered by several reviews

The included reviews examined a wide range of ICT interventions aimed at addressing social isolation and loneliness in older adults. Figure 4 provides a visual representation of the diverse array of technologies identified, which encompassed different functionalities and modes of engagement. The ICT interventions are classified into subcategories based on prior research [6], as well as emerging subcategories identified through data extraction from the current reviews. It is noteworthy that the identified categories are not mutually exclusive, as multiple reviews encompassed various technologies within each category. Consequently, the total number of technologies depicted in Fig. 4 exceeds the number of reviews included in this study. The following section presents examples of the different types of ICTs identified and their reported impact.

The social media ‘virtual communities’ emerged as the most frequently described digital technology across the included reviews [28, 39, 42, 44–48, 51, 53–59, 63–71]. This category includes participation in various support groups and social networking sites (SNSs), such as Facebook, MySpace, Google+, Xing, and WhatsApp. The majority of the reviews evaluating the impact of SNSs and other types of Internet-supported communication reported positive effects on reducing social isolation and loneliness [28, 45, 54, 55, 59, 63, 65, 67–71], as well as related outcome measures such as health-related quality of life and social connectedness [45, 75]. Although SNS use was largely associated with positive effects on loneliness and social isolation, some reviews reported mixed results and found the relationship to be inconclusive [39, 45, 51, 55, 59, 71]. It was noted that the positive effects were primarily short-term and did not persist for more than 6 months [45]. In addition, some studies reported negative effects, indicating that SNS users unintendedly experienced more loneliness than nonusers. In particular, one review highlighted that while direct communication via social networking sites was linked to decreased loneliness, passive engagement was associated with increased loneliness [68].

The use of video conferencing software to facilitate ‘friendly visits’ between older adults and their peers, family, friends, volunteers, staff, or healthcare professionals – commonly referred to as video-mediated friendly visits – involves the use of Internet and smartphone-based video calls, video chats, online games, and social media (e.g., FaceTime, Skype). Several reviews have assessed the effectiveness of these ICT solutions [37–39, 41, 42, 45, 50, 51, 58, 65, 66, 68, 71, 73]. Some reviews suggested that videoconferencing and Internet-based (supported) video communication were generally regarded positively by older adults and suggested positive impacts on social isolation [28, 39] and reductions in loneliness [37, 41, 71]; however, the evidence was based on a limited number of studies with limited quality.

The literature also included studies on the use of conversational agents with artificial intelligence (AI) functions (e.g., Care Coach, Tanya, AlwaysOn System) and social robots (e.g., Paro, Aibo, NAO, MARIO, iRobi, temi and animatronic, and robotic pets), aiming to engage users in dialogue for general socialization or to achieve specific health-related goals. Several of the reviews included in this study examined the impact of social robots or conversational agents in general [36–40, 46, 51, 52, 67], with the majority of results reported to provide some relief for loneliness [36, 37, 51, 52, 67] and indicated promising results in terms of social connectedness [39]. Some unintended negative consequences on social outcomes were also reported, such as sadness when the robot was removed [36]. The studies lacked sufficient data for informed conclusions [39], were found to be limited by small sample sizes and biases [67], and included very few randomized controlled trials [36, 51].

Several reviews examined the social effects of exergames (such as Nintendo Wii and Microsoft Xbox Kinect) and online gameplay [37, 38, 40, 42, 46, 47, 51, 74]. Augmented reality (AR) or virtual reality (VR) systems were covered by only one primary source in one of the included reviews [38]. Most reviews reported the effect of exergames [37, 42, 46] and online gameplay [40] in combination with the effect of a mix of ICTs. Nevertheless, three of the included reviews reported the specific effect of exergames and gameplay, indicating promising results regarding enhanced social well-being, including reductions in loneliness and social isolation [47, 51, 74]. In particular, studies of older adults with physical disabilities showed that playing exergames improved social well-being by increasing social bonding with peers and grandchildren [74]. However, the review of online gameplay only included one study examining loneliness, with contradictory results, suggesting that a higher frequency of gameplay was associated with increased social loneliness and reduced support from family and friends. Nonetheless, higher quality gameplay was associated with increased levels of social support [47].

Finally, it is important to highlight that the majority of reviews included in this study examined a combination of various ICTs and digital engagement interventions. These interventions encompassed sensor-based technology (e.g., smart home solutions), social networking sites, internet training, internet-delivered interventions, messaging services (e.g., e-mail, SMS), video chat, virtual spaces and classrooms with messaging capabilities, robotics, and games [38, 34, 35, 37–42, 45–52, 56–58, 61, 62, 64–68, 73, 75]. The inclusion of multiple interventions made it challenging to isolate the individual effects of each component. While some reviews concluded that a combination of ICTs can effectively provide social support to individuals [48, 56], the effectiveness of such interventions depends on the nature of the ICTs used and the individual’s well-being status [56]. Notably, one review cautioned that the use of ICTs could potentially have a negative impact on the quality of life for older adults, exacerbating feelings of loneliness [64]. This finding contrasts with the belief that technology use is essential for social engagement and combating loneliness [64]. Furthermore, several other reviews suggested that while ICTs can help older adults stay connected with their existing social networks, such as peers, family and friends, they generally do not contribute significantly to building new social connections [50, 55, 64]. Overall, the evidence suggests that the use of ICTs tends to reinforce existing social connections rather than facilitate the formation of new ones.

### Facilitators and barriers to the use of ICTs for reducing loneliness and social isolation

Several of the included reviews highlighted the facilitators and barriers associated with the use and implementation of ICTs aimed at reducing loneliness and social isolation. Based on the results and conclusions gathered from these reviews, we subcategorized these factors into three main groups: (i) individual competencies, encompassing digital literacy, health status, socioeconomic influences and demographic characteristics; (ii) access and usage, covering connectivity barriers, user interface challenges, digital learning opportunities and technological advancements; and (iii) intervention design and implementation, which included study design, relationship facilitation, ethical and social considerations, and healthcare system integration.

Regarding (i) individual competencies, limited technological knowledge, skills and unfamiliarity with technology were identified as barriers among older adults by several reviews [59, 65, 68]. Notably, training courses were identified as crucial facilitators for improving technological competence [47, 62, 70], whereas one review found that several studies provided moderate certainty evidence that internet training was associated with reduced loneliness [62]. One review showed that social support was found to increase when older adults spent more time using the Internet [34], and another review reported an increased adoption of more complex technological features among the elderly over time, with training and technical support interventions being seen as facilitators [73]. Conversely, the lack of such technical support and training interventions hindered progress [45, 49, 57, 66]. Socioeconomic factors [55, 57, 68] and health issues, including visual or hearing impairments, physical disabilities, and reduced coordination [45, 57, 59, 65, 74], were also identified as barriers. Furthermore, one review found that older individuals with more knowledge of the Internet, belonging to younger age groups, being women, and having fewer physical barriers experienced increased beneficial effects of ICTs [34].

Concerning (ii) access and usage, barriers to the adoption of ICTs for virtual communities among older adults included lack of Internet access [69] and language barriers in PC use [70]. Larger monitor screens and keytops were identified as facilitators for older adults with visual impairments [59]. For gameplay, the need for human and technological assistance was identified as a barrier [74]. Other reviews that focused on general or mixed ICT solutions raised similar concerns, such as challenges with sensor-based technology configurations [52] and accessibility issues to the Internet in general and to digital devices in particular [65, 68]. One review, examining how digital technology-based user interfaces can facilitate social interactions among older adults, identified four key factors influencing their social interaction experience though the interface. These factors were categorized as perceived usefulness, ease of use, accessibility and user preferences and behaviour [61].

Regarding (iii) intervention design and implementation, successful interventions were characterized by the facilitation of open communication, forming close relationships, ensuring shared experiences and characteristics, and inclusion of some form of pastoral guidance [28]. Another review highlighted that the effectiveness of ICTs in enhancing social connectedness depended on the study design and was improved by shorter durations, extended training periods, and the facilitation of preexisting relationships [39]. However, the lack of established and available social networks emerged as an important barrier [45, 50, 55, 64, 68], with ICTs proving effective for maintaining connections rather than expanding social networks. Remotely delivered interventions demonstrated effectiveness when they involved complex interactions with the individual, including empathy, intention, care, and attention, factors beyond the reach of medications alone or no intervention [41]. Furthermore, barriers to reducing loneliness or social isolation included challenges of accessibility, technology literacy, and intervention complexity [40]. Ethical challenges, including privacy, respect, and consent, were identified in several reviews [52, 57, 64, 65, 68]. The commitment and attitudes of healthcare providers toward technology in long-term care were also emphasized as influential factors [65].

### Identified research gaps in ICT interventions for reducing loneliness and social isolation

Several research gaps were identified in the use of ICT interventions to reduce loneliness and social isolation among older adults. These gaps include targeting specific subgroups, exploring innovative technologies, incorporating diverse study designs, improving research methodologies, and considering usability and accessibility.

Research should focus on identifying which elderly individuals can benefit the most from ICT use in reducing loneliness and social isolation [45]. Specifically, attention should be given to older adults at high risk of experiencing loneliness in daily life, such as the oldest-old population and homebound individuals [50, 54]. Studies should also encompass older adults across various settings [76] and evaluate the impact of interventions on informal family caregivers [68, 73]. Customized digital technologies tailored to users’ specific contextual and individual characteristics should be tested in future research [51, 66]. Additionally, vulnerable populations, including the oldest-old, ethnic or sexual minorities, low socioeconomic status individuals, those from low-income countries with limited access to digital technologies, and individuals with low health literacy, should be targeted [58].

To broaden the understanding of technology-based interventions, research should explore more innovative technologies [50]. This includes virtual and augmented reality applications, machine learning and artificial intelligence-powered virtual assistants [39, 44, 56, 66], mHealth interventions [67], exergaming platforms [74], the effect of smartphone-based instant messaging applications [56], video calls [76], digital technologies and service models with a broader social perspective [61] and diverse technological training solutions [57, 62, 73].

To capture the complexity of loneliness, social isolation and social participation, future studies should include a wider range of study designs, incorporating both qualitative and quantitative data [48, 50]. High-quality randomized controlled trials with larger sample sizes and longitudinal designs are recommended to better understand causal mechanisms and provide more robust conclusions [36, 38–41, 50, 58, 63, 70]. Attention should also be given to the development of appropriate research instruments [38, 64], particularly those that capture the distinct yet related concepts of social isolation and loneliness [66]. Triangulation of loneliness measures, including observations, validated self-report measures, and interviews, can provide deeper insights into effects [36].

Future studies should address the usability, affordability and accessibility of ICT interventions [46], taking into account the needs and values of older adults in technology design [42]. Matching older adults with technology interventions that align with their interests can improve health outcomes [57]. Perceived behaviours and self-directed use of off-the-shelf communication technologies by older adults to overcome loneliness and social isolation should be further explored [38]. Participatory and human-centred designs should be prioritized for the development of future technologies tailored to older adults, with a focus on sustainability [38].

## Discussion

A scoping review of reviews was conducted to examine the literature on ICT interventions aimed at reducing loneliness and social isolation among older adults living in the community. This review aimed to provide an overview of the different types of ICT interventions, their impact, and the facilitators and barriers associated with their use. Additionally, the review sought to identify gaps in the existing research and to summarize the suggested areas for future investigation. In the following section, a summary of the main findings is presented. As several interesting findings emerged from the included research literature, we will emphasize and discuss some of the key aspects.

### Summary of main findings

This scoping review of reviews included 39 publications with varying characteristics. The majority of reviews were systematic reviews, scoping reviews, or reviews of reviews published between 2012 and 2024, with a significant increase in publications from 2020 to 2021. As the relevance of studying loneliness and social isolation among older adults increased during the COVID-19 pandemic [40], this may explain the increased number of published reviews in this field of interest after 2020. Furthermore, definitions of “older adults” and gender distribution were inconsistent among the reviews. The studies covered different settings, including older adults solely living at home or combined with those in nursing homes or residential care facilities. Some reviews focused on interventions to reduce social isolation or loneliness, while others covered both. A wide range of ICTs were examined, with social media virtual communities being the most commonly described technology. Positive effects on reducing social isolation and loneliness were reported for social media virtual communities, video-mediated friendly visits, conversational agents, and social robots. Exergames and online gameplay had positive effects on social well-being but inconsistent results regarding loneliness. The use of multiple ICTs made it difficult to determine their individual effects. It was not within the scope of this review to assess the methodological quality of individual reviews included in the analysis. However, it is worth noting that several reviews identified the majority of included studies as having low to moderate quality and with significant limitations. Individual competencies, access and usage, and intervention design and implementation were identified as facilitators and barriers to the use of ICTs. Research gaps included targeting specific subgroups, exploring innovative technologies, incorporating diverse study designs, improving research methodologies, and addressing usability and accessibility issues. Future research should focus on identifying elderly individuals who can benefit the most from ICT use, exploring new technologies, using a wider range of study designs, and improving usability and accessibility considerations.

### Challenges in defining ‘older adults’ and implications for ICT interventions

The substantial variation in the definition of ‘older adults’ across the included reviews and primary studies complicates the comparability and generalizability of findings across studies. The inclusion of participants as young as 45 years old [68] or encompassing a broad age range from children to older adults [56, 75] raises concerns regarding the applicability of the findings to the specific target population for whom the ICT interventions are intended. The different age thresholds likely result in variations in the characteristics and needs of the participants, which can impact the effectiveness and relevance of ICT interventions in reducing social isolation and loneliness. Additionally, the varying age thresholds may also affect the feasibility and acceptability of ICT interventions among different age groups. Rather than categorizing respondents into broad age groups, the use of continuous measures has been suggested [77]. This approach may provide a more nuanced understanding of the specific implications of ICTs at different stages of life. Furthermore, the perception of ‘older adults’ is context-dependent and evolves over time, varying across communities, individuals, and cultures [78, 79]. Factors such as subjective, biological, physiological, and social age contribute to this dynamic view. Societal norms, healthcare, and cultural influences further shape this context, leading to diverse definitions of age. This complexity holds particular significance for technological interventions aimed at older adults. Recognizing these nuances, we suggest that age perceptions are crucial for designing effective ICT interventions that cater to the diverse needs of older populations globally.

### Considerations for outcome measures and gender differences

Careful consideration and selection of outcome measures is crucial to advance research on social isolation and loneliness among older adults. Foremost, the use of appropriate assessments is critical, and although social isolation and loneliness are concepts that have been defined in various ways, several studies included in the current review commonly used outcome measures capturing elements of both. While assessing loneliness and social isolation in combination may seem effective in clinical trials, it is crucial to select the appropriate assessment tool that aligns with the research question or intervention being studied to avoid confusion between the two concepts [66, 80]. Unfortunately, a significant barrier is the absence of a standardized, internationally recognized, and cross-culturally valid measure of these two concepts, despite the myriad of instruments available for measuring social isolation and loneliness [80, 81]. Additionally, using validated measurement tools will help to build a more robust evidence base, and efforts should be made to update existing measures and develop better instruments that capture the experience of today’s older adults [64], including newer modes of communication such as social media and video conferencing. Furthermore, it is essential to conduct longitudinal testing because social isolation and loneliness can fluctuate over time. To reliably measure trajectories, standardized assessment tools should be consistently used at all time points. Only a limited number of reviews [51, 55] included in this study reported the inclusion of primary studies with a longitudinal design.

Research also suggests that men and women may have different understandings and interpretations of loneliness. While loneliness is a universal human experience, societal and cultural factors can influence how individuals perceive and express their loneliness. Studies have shown that older women often report higher levels of loneliness than older men [82, 83]. This difference could be attributed to various factors, including gendered social roles, socialization patterns, and communication styles. It has also been proposed that men are more likely to underreport being lonely when direct measures of loneliness are used [84]. Another study highlighted that gender differences in loneliness are highly dependent on the assessment mode used and whether demographic and psychosocial differences between men and women are taken into account [77]. By delving deeper into the distinct perspectives and experiences of men and women, tailored approaches and outcome assessments can be developed to address the specific needs and challenges faced by each gender in combatting loneliness.

### The paradox of the negative impact of ICT interventions

While the majority of ICT interventions have shown promise in reducing social isolation and loneliness, there exists a paradoxical phenomenon where these interventions can sometimes have a negative impact on the well-being of older adults, especially among those who are already socially isolated or lonely [45]. Our review highlights that ICTs involving social networking sites and virtual communities are particularly prone to these unintended consequences. It may therefore be essential to consider these ICTs as supplementary to in-person social interactions rather than as substitutes [45, 54]. One review also emphasizes the insufficient attention given to the potential negative emotions that digital technologies may evoke in older adults [61] Moreover, our findings underscore the importance of targeting interventions towards older adults who lack social networks rather than solely focusing on those who are already socially connected. By addressing the specific needs of lonely and socially isolated older adults, such as enabling open communication and fostering close relationships, improved social support could be achieved [28]. Future research should further investigate the potential negative impact of SNS use on this vulnerable population and develop interventions that effectively establish new social networks and mitigate the adverse effects of technology use.

### Overcoming barriers and exploring facilitators for older adults

Several reviews included in this study highlight the numerous barriers and challenges that hinder the utilization of ICTs among older adults. These barriers arise from individual competencies, access and usage, and intervention design and implementation, as summarized in the results section. Understanding and overcoming these barriers are crucial to determine the effectiveness of technological solutions for individual older adults in specific contexts. However, the existence of various barriers raises an important question: should ICT interventions conform to the requirements and difficulties of older adults, or is it expected that older adults adapt to the technology?

One of the reviews included in this analysis, offering insights into recent developments in research on user interfaces that support social interaction among older adults, underscores the intricate factors influencing the effects of user interfaces, including perceived usefulness, ease of use, accessibility, user preferences and behaviours [61]. To enable the effective adoption and use of technology, Mannheim and colleagues (2019) emphasize the involvement of older adults in the design process and research of digital technologies [85]. They argue that ageism, characterized as biased knowledge, values, attitudes and behaviours towards older people, may be a significant barrier to technology adoption. Therefore, addressing ageism becomes imperative to ensure successful implementation.

In addition, it is recommended that further qualitative research explore the criteria for successful technological solutions among older adults. It is important to consider the facilitators that can enable the successful adoption and use of technology. While the lack of digital competence among the elderly is commonly reported as a barrier, technological training interventions have been found to effectively improve digital skills and confidence in older adults [23] and reducing loneliness [62]. However, it is worth noting that technological training interventions have received less attention in the included reviews. This indicates a need for more research to evaluate the effectiveness of such interventions, which have the potential to address the lack of technological competence and enable successful adoption of technological solutions among older adults [86].

### Future research

Consensus was reached among the majority of the included reviews regarding the need for further research on the development, impact and implementation of ICTs on loneliness and social isolation in older adults. In line with the comprehensive scoping review of reviews conducted by Fakoya and colleagues (2020), which covered a wide range of both technological and nontechnological interventions, future studies should focus on identifying which ICT interventions work for whom, in what particular context, and how [9]. To achieve these goals, future research should prioritize the identification of older individuals who can benefit the most from ICT use. It is also important to explore innovative technologies and employ a broader range of high-quality study designs to gather robust evidence. Attention should be given to the development of appropriate research instruments, in addition to employing triangulation of loneliness measures to provide deeper insight into effects and enhance the understanding of causal mechanisms. Improving usability, affordability and accessibility considerations should be a key focus to ensure that the technologies are user-friendly and accessible to older adults.

### Study strengths and limitations

Scoping reviews are useful tools for identifying and summarizing existing evidence and identifying research gaps on a specific research question [29]. However, it is important to consider its limitations when interpreting the findings. One limitation of this scoping review is that it focused solely on ICTs, which may not fully represent the broader field of gerontechnology. While ICTs are a significant component of gerontechnology, other technological interventions might also play a crucial role in addressing loneliness and social isolation for older adults. Another limitation is related to the participant characteristics and settings included in the review. We primarily focused on community-dwelling older adults living at home or in combination with those living in long-term care facilities. While this diverse representation provides a broad understanding of intervention effectiveness and implications across different contexts and populations within the older adult demographic, the findings may not be generalizable to older adults with specific medical diagnostic challenges living in long-term care facilities or in hospital or rehabilitation settings.

The inclusion of older people across a large age range is another limitation that may introduce heterogeneity and make it challenging to draw specific conclusions. However, this reflects the nature of the research field, where the definition of the older adult population is diverse and spans a wide age range. Despite this challenge, efforts should be made to account for age-related differences in future studies.

Categorizing ICT interventions into mutually exclusive subcategories was challenging due to inconsistent terminology and the lack of standardized frameworks. Additionally, the heterogeneity of interventions and the variety of outcome measures limit the ability to draw strong conclusions about the effectiveness of different interventions. Finally, our search strategy included a limited number of keywords, which may have resulted in the omission of relevant literature. Despite our efforts to compensate for this limitation by employing backwards and forward citation chaining, it is still possible that important reviews were missed. Future research should consider a more comprehensive and refined search strategy to ensure more exhaustive coverage of the literature.

## Conclusions

In conclusion, this scoping review of reviews offers valuable insights into the landscape of ICT interventions targeting the reduction of loneliness and social isolation among older adults living in the community. The review sheds light on the diverse range of ICTs, their impact, and the facilitators and barriers associated with their use. It also underscores the challenges arising from the varying definitions of ‘older adults’ across reviews, which challenge comparability and generalizability. The paradoxical phenomenon of potential negative impacts of ICT interventions on already socially isolated individuals highlights the need for cautious implementation. Methodological limitations and gaps identified within the literature encourage future investigations to prioritize refining outcome measures, addressing gender differences, and enhancing the usability and accessibility of ICT interventions. The involvement of older adults in the design process and the exploration of technological training interventions hold promise in overcoming barriers. By pursuing these possibilities, researchers may better tailor ICT interventions to the specific needs of older adults.

### Supplementary Information

**Additional file 1:.** Search strategy

**Additional file 2:.** Evidence table overview: Characteristics of included reviews (N=39), sorted by year of publication

## Data Availability

The datasets used and/or analysed during the current study are available from the corresponding author on reasonable request.

## Abbreviations

AR: Augmented reality
ICT: information and communication technology
JBI: Joanna Briggs Institute
OECD: Organization for Economic Co-operation and Development
PCC: population-concept-context
PRISMA: The Preferred Reporting Items for Systematic Reviews and Meta-Analyses
SNS: social networking site
UCLA: University of California Los Angeles Loneliness scale
VR: virtual reality
WHO: World Health Organization
