# Supplementary material for: Exploring the impact of information and communication technologies on loneliness and social isolation in community-dwelling older adults: a scoping review of reviews
Source: BMC Geriatr. 2024 Mar 2;24:215. doi: 10.1186/s12877-024-04837-1 (PMC10908002; doi:10.1186/s12877-024-04837-1)
Supplement: Supplementary file 1 — Additional file 1:. Search strategy [file 12877_2024_4837_MOESM1_ESM.docx]

**APPENDIX 1.**

Original search strategy

**PubMed**

16.08.2022

| # | Search | Results |
| --- | --- | --- |
| #1 | (elderly OR "old adults" OR aged OR aging OR senior) AND (loneliness OR "social isolation" OR "social participation") AND ("digital technology" OR "ICT intervention" OR e-intervention OR internet OR "social media") | 608 |
| #2 | #1 and **review article** (document type) | 40 |

**Scopus**

16.08.2022

| # | Search | Results |
| --- | --- | --- |
| #1 | (elderly OR "old adults" OR aged OR aging OR senior) AND (loneliness OR "social isolation" OR "social participation") AND ("digital technology" OR "ICT intervention" OR e-intervention OR internet OR "social media") | 1157 |
| #2 | #1 and **review article** (document type) | 89 |

**Medline**

16.08.2022

| # | Search | Results |
| --- | --- | --- |
| #1 | (elderly OR "old adults" OR aged OR aging OR senior) AND (loneliness OR "social isolation" OR "social participation") AND ("digital technology" OR "ICT intervention" OR e-intervention OR internet OR "social media") | 566 |
| #2 | #1 and **review article** (document type) | 34 |

**Cochrane Library**

16.08.2022

| # | Search | Results |
| --- | --- | --- |
| #1 | (elderly OR "old adults" OR aged OR aging OR senior) AND (loneliness OR "social isolation" OR "social participation") AND ("digital technology" OR "ICT intervention" OR e-intervention OR internet OR "social media") | 136 |
| #2 | #1 and **Cochrane reviews** (document type) | 1 |

**Web of Science**

18.08.2022

| # | Search | Results |
| --- | --- | --- |
| #1 | (elderly OR "old adults" OR aged OR aging OR senior) AND (loneliness OR "social isolation" OR "social participation") AND ("digital technology" OR "ICT intervention" OR e-intervention OR internet OR "social media") | 1162 |
| #2 | #1 and **review article** (document type) | 63 |

**PsychINFO**

18.08.2022

| # | Search | Results |
| --- | --- | --- |
| #1 | (elderly OR "old adults" OR aged OR aging OR senior) AND (loneliness OR "social isolation" OR "social participation") AND ("digital technology" OR "ICT intervention" OR e-intervention OR internet OR "social media") | 358 |
| #2 | #1 and **review article** (document type) | 26 |

**CINAHL**

18.08.2022

| # | Search | Results |
| --- | --- | --- |
| #1 | (elderly OR "old adults" OR aged OR aging OR senior) AND (loneliness OR "social isolation" OR "social participation") AND ("digital technology" OR "ICT intervention" OR e-intervention OR internet OR "social media") | 559 |
| #2 | #1 and **review article** (document type) | 15 |

**Epistemonikos**

18.08.2022

| # | Search | Results |
| --- | --- | --- |
| #1 | (elderly OR "old adults" OR aged OR aging OR senior) AND (loneliness OR "social isolation" OR "social participation") AND ("digital technology" OR "ICT intervention" OR e-intervention OR internet OR "social media") | 255 |
| #2 | #1 and **systematic** **review** (document type) | 28 |

Updated search strategy

**PubMed**

13.02.2024

| # | Search | Results |
| --- | --- | --- |
| #1 | (elderly OR "old adults" OR aged OR aging OR senior) AND (loneliness OR "social isolation" OR "social participation") AND ("digital technology" OR "ICT intervention" OR e-intervention OR internet OR "social media") | 778 |
| #2 | #1 and **review article** (document type) | 53 |
| #3 | #2 and 16.08.2022 – today (publication date) | 14 |

**Scopus**

13.02.2024

| # | Search | Results |
| --- | --- | --- |
| #1 | (elderly OR "old adults" OR aged OR aging OR senior) AND (loneliness OR "social isolation" OR "social participation") AND ("digital technology" OR "ICT intervention" OR e-intervention OR internet OR "social media") | 1500 |
| #2 | #1 and **review article** (document type) | 105 |
| #3 | #2 and limited to 2022, 2023, 2024 | 39 |

**Medline (Ebscohost)**

13.02.2024

| # | Search | Results |
| --- | --- | --- |
| #1 | (elderly OR "old adults" OR aged OR aging OR senior) AND (loneliness OR "social isolation" OR "social participation") AND ("digital technology" OR "ICT intervention" OR e-intervention OR internet OR "social media") | 858 |
| #2 | #1 and **review article** (document type) | 55 |
| #3 | #2 and August 2022 – today (publication date) | 14 |

**Cochrane Library**

13.02.2024

| # | Search | Results |
| --- | --- | --- |
| #1 | (elderly OR "old adults" OR aged OR aging OR senior) AND (loneliness OR "social isolation" OR "social participation") AND ("digital technology" OR "ICT intervention" OR e-intervention OR internet OR "social media") | 197 |
| #2 | #1 and **Cochrane reviews** (document type) | 1 |
| #3 | #2 and Cochrane reviews (document type) | 0 |

**Web of Science**

13.02.2024

| # | Search | Results |
| --- | --- | --- |
| #1 | (elderly OR "old adults" OR aged OR aging OR senior) AND (loneliness OR "social isolation" OR "social participation") AND ("digital technology" OR "ICT intervention" OR e-intervention OR internet OR "social media") | 1521 |
| #2 | #1 and **review article** (document type) | 91 |
| #3 | #2 and limited to 2022, 2023, 2024 | 43 |

**PsychINFO**

*Database search not updated. No longer accessible from job institution.*

**CINAHL**

13.02.2024

| # | Search | Results |
| --- | --- | --- |
| #1 | (elderly OR "old adults" OR aged OR aging OR senior) AND (loneliness OR "social isolation" OR "social participation") AND ("digital technology" OR "ICT intervention" OR e-intervention OR internet OR "social media") | 748 |
| #2 | #1 and **review article** (document type) | 15 |
| #3 | #2 and August 2022 - today (publication date) | 1 |

**Epistemonikos**

13.02.2024

| # | Search | Results |
| --- | --- | --- |
| #1 | (elderly OR "old adults" OR aged OR aging OR senior) AND (loneliness OR "social isolation" OR "social participation") AND ("digital technology" OR "ICT intervention" OR e-intervention OR internet OR "social media") | 422 |
| #2 | #1 and **systematic** **review** (document type) | 43 |
| #3 | #2 and limited to 2022, 2023, 2024 | 22 |
