# Supplementary material for: Exploring the impact of information and communication technologies on loneliness and social isolation in community-dwelling older adults: a scoping review of reviews
Source: BMC Geriatr. 2024 Mar 2;24:215. doi: 10.1186/s12877-024-04837-1 (PMC10908002; doi:10.1186/s12877-024-04837-1)
Supplement: Supplementary file 2 — Additional file 2:. Evidence table overview: Characteristics of included reviews (N=39), sorted by year of publication [file 12877_2024_4837_MOESM2_ESM.docx]

APPENDIX 2. Evidence table overview: Characteristics of included reviews (N=39), sorted by year of publication.

| Author(s), (year of publication)  *Title*  Journal | Type of review (number of included studies) | Aims and objective of review | Population (age, gender) | Study designs included | Type of ICT intervention(s) / technology focus | Outcome assessments | Key results |
| --- | --- | --- | --- | --- | --- | --- | --- |
| Choi, Kong, Jung (2012)  *Computer and Internet Interventions for Loneliness and Depression in Older Adults: A Meta-Analysis*  Healthcare Informatics Research | Meta-analysis (6 studies) | This study conducted a meta-analysis to examine the effectiveness of computer and Internet training interventions intended to reduce loneliness and depression in older adults. | Older adults from 64 to 83 years. Gender not specified. | Randomized controlled trials (n=4), quasi-experimental design (n=2). | All interventions included both computer and Internet training (no further specification of type of intervention), and 4 studies provided computers as well to the older adults they studied. | Loneliness assessed with: UCLA loneliness scale, revised UCLA loneliness scale, de Jong Gierveld loneliness scale. | The overall mean weighted effect size for loneliness in the five studies was 0.546 with a 95% CI of 0.033-1.059 (Z = 2.085, p = 0.037) based on data from 353 older adults. Thus, computer and Internet training interventions had an effect in decreasing loneliness in older adults. They found that these interventions were significantly effective in decreasing loneliness, but ineffective in decreasing depression. Therefore, further computer-mediated social support should be considered to help manage loneliness in this population. |
| Nef, Ganea, Müri, Mosimann (2013)  *Social networking sites and older users - A systematic review*  International Psychogeriatrics | Systematic review (18 studies) | Review, classify, and summarize existing scientific literature. Discuss possible negative and positive impacts of social networking sites (SNS) use on mental health and present possible future research directions. | Field tests: older adults from 55 to 97 years, sample size ranged from 1 to 60. User groups, interviews, and online surveys: older people ranged from 50 to >80 years, sample size ranged from 5 to 3500. Gender not specified. | 5 articles with results from field tests, 13 articles with results from focus groups, questionnaires, and interviews. | Field tests: SNS, touchscreen-based interface for communication with SNS / graphical user interface, technological integrations in Facebook. Focus groups, questionnaire, interviews: explored participants’ perceptions of and stories about computers, general technological involvement, older adults’ thoughts and feelings toward SNS, analysed the reasons for the low uptake of SNS by older adults, older adults’ perceptions of SNS, and what educational strategies can facilitate older adults’ learning of SNS, examined the influence of Internet use on communication and social involvement, interviews around daily routines and activities, communication patterns, and technology and social media usage, weekly Facebook use on social capital, why people do (not) use particular online services, focusing on the influence of collective self-esteem and group identity on motives for SNS use, SNS use and four social capital dimensions: face-to-face interactions, number of acquaintances, and bridging capital. | Not specified - much of the reported results are qualitative analyses of interviews and observations from different perspectives. No specific outcome assessment mentioned in review. | The main benefit of using social networking sites for older adults is to enter in an intergenerational communication with younger family members (children and grandchildren) that is appreciated by both sides. Identified barriers are privacy concerns, technical difficulties and the fact that current Web design does not take the needs of older users into account. Two studies specifically focused on loneliness (Eggermont 2009, Brantzæg 2012):  Eggermont (2009) found mixed results from focus groups - older people would like to see SNS to support the social relationships of older adults and help fight loneliness, but older adults also strongly plead for the maintenance of non-mediated communication (face-to-face) and non-technological alternatives.  Brandtzæg (2012) found that SNS-users reported more loneliness than non-users, suggest that the usage of SNS and social contact are supplementary, and SNS use might extend existing levels of social contact in all age groups. |
| Morris, Adair, Kurowski, Miller, Pearce, Santamaria, Long, Ventura, Said (2014)  *Smart technologies to enhance social connectedness in older people who live at home*  Australasian journal on ageing | Systematic review (18 studies) | The aim of this study was to undertake a systematic literature review of studies that assessed the effectiveness of smart technologies in improving or maintaining the social connectedness of older people living at home. | Not defined (n=13), 40-70 years (n=1), 17-51 years (n=1), 11-51 years (n=1), 30-69 years (n=1), 57-85 years (n=1). Gender not specified. | RCT (n=12), cohort studies (n=6) | Web-based information, intervention, and communication programs. Approximately half of the studies incorporated an online education program that provided information related to the health condition of interest. A small number also provided email access to health professionals. Three studies provided participants with the necessary equipment as well as training for computer, Internet and email use. Other interventions included the use of pre-recorded, interactive telephone messages, the Nintendo Wii, and an automated, online self-help program. Only one study provided visual and verbal contact between study participants. | Fifty-eight different outcome indicators of social connectedness were utilised. Of these, the most frequently used measures were the Center for Epidemiological Studies Depression Scale (nine studies), the University of California Los Angeles (UCLA) Loneliness Scale (four studies) and the Rosenberg Self-Esteem Scale (four studies). | Variable results were reported regarding the effect of smart technologies on dimensions of social connectedness. Overall, the most consistent results were for studies that addressed social support: six studies reported that the use of smart technology over periods of less than one year could help to achieve statistically significant improvements in this construct. Some of the studies that assessed aspects of social support also included outcome measures of empowerment, loneliness and social networks. Three out of five studies found positive results for empowerment when using interactive, online programs that incorporated health-based information, chat rooms and discussion forums. In relation to the constructs that are potentially related to social connectedness, it appeared that smart technologies (predominantly interactive, online programs and discussion forums) had some positive effect on quality of life and health related quality of life. |
| Hagan, Manktelow, Taylor, Mallett (2014)  *Reducing loneliness amongst older people: a systematic search and narrative review*  Aging & Mental Health | Narrative review (17 articles, whereas six studies investigated the use of new technologies in possibly reducing loneliness). | To investigate the effectiveness of recent social therapeutic interventions to reduce loneliness in older people. | Technology-based studies: Aged from 55+ to 94. Gender: female > male. | Technology-based studies: Controlled trial/RCT (3), pilot/exploratory study (1), evaluation/post-hoc study (1), before and after-design (1). | Internet-based video communication, web-based communication, Internet-usage, game console (Nintendo Wii), animal-assisted therapy (robotic dog). | Three of the six included studies assessing new technologies used UCLA Loneliness scale, others not specified. | Three studies reporting on new technologies (i.e., Nintendo Wii, robotic dog, videoconferencing) and one on a group work intervention identified significant reductions in loneliness. This finding would seem to support future development of the use of innovative interventions and perhaps helps quash perceptions that older people are resistant to new technologies. |
| Chen & Schulz (2016)  *The Effect of Information Communication Technology Interventions on Reducing Social Isolation in the Elderly: A Systematic Review*  Journal of Medical Internet Research | Systematic review (25 publications) | The objective of this systematic review is to gain a synthesis of the evident effects of ICT interventions on social isolation in the elderly. | Elderly (average age ranged from 66 years to 83 (SD 1.4) years), Gender: mixed (n=22), n/a (n=3). | 25 publications describing 30 studies: 6 RCTs, 6 cohort studies, 4 cross-sectional studies (surveys), 14 qualitative studies (including 9 with in-depth interviews, 3 with focus-group interviews, 2 participant observation). | Information and communication technologies (ICT): Most research used some form of Internet or Web-based apps (e.g., search, email, online chat rooms, videoconferencing, social networking apps, and Web-based telehealth systems) on computers. Among those that did not, 1 study employed a telephone befriending intervention, 1 used mobile phones (smartphones), 1 focused on iPad use, 1 applied Nintendo Wii (a video game system), and 1 used a visual pet companion app that allowed the senior users to interact with a pet avatar in real time through an android tablet. | Assessing the effectiveness of ICT-mediated social isolation interventions for elderly. Social isolation assessed by one self-developed scale, otherwise not defined. Loneliness was assessed by the University of California Los Angeles (UCLA) Loneliness Scale in 20 of 25 projects. Other scales used: Jong-Gierveld and Kamphuis' loneliness scale (long and short version), social support scale by Schuster et al, Social and Emotional Loneliness Scale, and self-reported items of loneliness by elderly. Social support assessed by Hsiung's Social Support Behaviours Scale, and Family and Friendship Contacts Scale. Social well-being assessed by de Jong-Gierveld and Kamphuis' loneliness scale. | ICT use was consistently found to affect social support, social connectedness, and social isolation in general positively. The results for loneliness were inconclusive. Even though most were positive, some studies found a nonsignificant or negative impact. More importantly, the positive effect of ICT use on social connectedness and social support seemed to be short-term and did not last for more than six months after the intervention. |
| Fan (2016)  *Utilizing ICT to prevent loneliness and social isolation of the elderly. A literature review*  Cuadernos de Trabajo Social | Literature review (34 articles) | The aim of this literature review is to map out the state of the art of knowledge about the usage of ICT in elderly care and to figure out research-based knowledge about the usability of ICT for the prevention of loneliness and social isolation of elderly people. | Different age groups, mainly represented by 55+. Two articles with participants from young adults to old adults. Gender not specified. | Quantitative data (n=20), qualitative data (n=10), mix of both quantitative and qualitative data (n=3). | ICT-based solutions. | Not specified. | The results show that the so-called ‘digital divide’ still exists, but older adults have the willingness to learn and utilize ICT in daily life, especially for communication. The data shows that the usage of ICT can prevent loneliness and social isolation of older adults, and they are eager for technical support in using ICT. The results of data analysis on theoretical frames and concepts show that this research field applies different theoretical frames from various scientific fields, while a social work approach is lacking. However, a synergic frame of applied theories will be suggested from the perspective of social work. |
| Khosravi, Rezvani, Wiewiora. (2016)  *The impact of technology on older adults' social isolation*  Computers in Human Behavior | Systematic literature review (34 papers) | This systematic review aims to identify ICTs that are designed to help seniors reduce their social isolation and loneliness and assess the effectiveness of this technology in supporting seniors’ wellbeing. | Seniors aged 50 or older (large range, some are among the oldest old). Less information regarding gender. | RCT (n=5),  quasi-experimental study (n=3), survey-cross-sectional (n=4), survey-longitudinal (n=2) | General ICT (n=15), Robotics (n=8), SNS (n=3), video game (n=1), 3D virtual environment (n=1), PRISMS (n=1), Health support chat room (n=1) | Most of the studies used validated measurement tools, with the University of California Los Angeles (UCLA) loneliness scale being used in the greatest number of studies. de Jong Gierveld and Havens’ (2004) loneliness scale, developed to measure loneliness in older people, was also used in a few studies. | The study identified eight different technologies that have been used with seniors and assessed their effectiveness. Findings from this study show that various technologies offer different possibilities and ways of engagement and, generally, most of them can be used to reduce social isolation and loneliness among seniors. |
| Chipps, Jarvis, Ramlall (2017)  *The effectiveness of e-Interventions on reducing social isolation in older persons: A systematic review of systematic reviews.*  Journal of telemedicine and telecare | Systematic review of systematic reviews (i.e., reviews that used systematic review methodology to review quantitative comparative studies) (12 reviews) | Review question: What is the level of evidence on the effectiveness of eInterventions to reduce social isolation and loneliness in older people living in community/residential care? | Elderly (age ranged from 50 - 105). Gender: mixed (n=11), n/a (n=1). | 12 reviews (8 systematic reviews without meta-analysis, 3 meta-analyses, 1 integrative review), compiling 22 unique primary research studies (i.e., 6 RCTs, 8 quasi-experimental studies, 8 cross-sectional studies). | **eInterventions** (i.e., interventions that employ any ICT or Internet-supported intervention delivery mode with or without human support) to reduce social isolation or loneliness in older people living in community/residential care. The most common eIntervention reported was ‘online activities’ such as computer/Internet training and usage (n=15). In this review the interventions were classified into online activities, interpersonal, Internet-supported communications and Internet-supported therapeutics such as robotics, games and relational agents. | Quantitative outcome data: Loneliness most frequently assessed by different versions of the University California Los Angeles (UCLA) Loneliness Scale. European studies tended to select versions of the de Jong Gierveld Loneliness Scale (DJGLS). | Training and use of Internet/computer eInterventions were not supported with conclusive evidence on the impact on loneliness. The evidence for Internet-supported communication showed a significant reduction in loneliness, though this was mediated by self-efficacy and frequency of use. The emergence of the field of robotics showed some evidence of potential for decreasing loneliness, though the studies were small and biased. |
| Damant, Knapp, Freddolino, Lombard (2017)  *Effects of digital engagement on the quality of life of older people*  Health and Social Care in the Community | Scoping review (including academic and grey literature, N=91 key articles) | To review evidence on the impact of ICT on quality of life (QOL) of older people, with respect to both their day-to-day lives in general and their health and social care needs in particular. | Total sample: Older persons (aged 50 to 84). Age span in loneliness-studies: 57+/ 60+/50+/64-75/55+/60+. Gender: n/a | A wide range of both qualitative and quantitative research methods and instruments are employed across many studies with varying effects. | Mainstream ICTs were defined to be contemporary generic technology devices, services, applications and Internet platforms used by large proportions of the population in Britain as indicated by OfCom (2014). It includes Internet networks, mobile phones, smart phones, computers and tablet computers. Technology reported in result-table specific to loneliness were ICT training, alarms and monitoring, Skype, email, computer training (and use), internet, videoconferencing services. | Different types of quality of life (QOL) instruments. Specific outcomes assessing loneliness were UCLA Loneliness scale, Social and Emotional Loneliness scale, De Jong Gierveld and Van Tilburg, study-specific instruments. Social support assessments: Social Support Appraisal Scale. | Overall results: The review revealed mixed results. Older people’s use of ICT in both mainstream and care contexts has been shown to have both positive and negative impacts on several aspects of QOL. Studies which have rigorously assessed the impact of older people’s use of ICT on their QOL mostly demonstrate little effect. A number of qualitative studies have reported on the positive effects for older people who use ICT such as email or Skype to keep in touch with family and friends. Specific results regarding loneliness: Overall the results from qualitative studies suggested that ICT use had positive impacts on family contacts and intergenerational relationships. Weak, negative or insignificant effects on loneliness. Visiting and general social functioning were most often reported in quantitative studies. Some findings implied that ICT use could negatively affect QOL by exacerbating feelings of loneliness. Therefore, contrary to the assumption that engagement with ICT is essential for older people to remain socially active and to combat loneliness, the evidence could be said to imply that use of ICT positively reinforces existing social networks, but generally has no effect on building new ones. |
| Baker, Warburton, Waycott, Batchelor, Hoang, Dow, Ozanne, Vetere (2018)  *Combatting social isolation and increasing social participation of older adults through the use of technology: A systematic review of existing evidence*  Australasian journal on aging | Systematic review (36 papers) | The aim of this systematic review was to investigate how technology is being used to combat social isolation and increase social participation for older adults. | Age-group included not specified. According to inclusion criteria: older people (i.e., the term as used in the literature, although generally identifies as aged over 65 years). Gender: N/A. | 16 interview-based qualitative evaluations, 8 smaller scale designs, pilot and/or prototype evaluations, 2 surveys, 3 mixed-method evaluations, 2 social network analyses, 5 quantitative evaluations. | Touch-screen technology, social network services (SNS), adaption of use of existing technology platforms, use of games to encourage social participation, ICT training analyses and interview studies. | The studies include a range of outcomes measures. The range of social concepts utilised in the review literature varied considerably, with many failing to define clearly the social outcomes being addressed in their study, whether it related to redressing social isolation or increasing social participation. | Findings were threefold, suggesting that: (i) technologies principally utilised social network services and touch-screen technologies; (ii) social outcomes are often ill-defined or not defined at all; and (iii) methodologies used to evaluate interventions were often limited and small-scale. |
| Li, Erdt, Chen, Cao, Lee, Theng (2018)  *The social effects of exergames on older adults: Systematic review and metric analysis*  Journal of medical internet research | Systematic review (10 studies) | The aim of this paper is to synthesize existing studies and provide an overall picture on the social effects of exergames on older adults. | Participants aged 55 and above. Mean age 80 year and up (n=3), mean age 70-80 years (n=1), mean age 60-69 years (n=1), range from 56-92 year (n=1), ˃ 60 year (n=1), ˃ 55 year (n=1), 75 year (n=1), and not available (n=1). Gender not specified. | Mix of qualitative and quantitative studies. | Nintendo Wii Sports (n=8) and Kinect Sports (n=2). | UCLA Loneliness Scale (n=3). Semi structured interview (n=3), Focus group discussion (n=1), Interview (n=1), Semi structured group interview (n=1) | Most of the studies recruited healthy older adults from local communities or senior activity centres. Three groups of social-related outcomes have been identified, including emotion-related, behaviour-related, and attitude-related outcomes. A metric analysis has shown that the emotion-related and behaviour-related outcomes received high attention from both the academic community and social media platforms. The two included studies with older adult participants with physical disabilities showed that playing exergames improved their social well-being by increasing social bonding with their peers and grandchildren. Jung et al conducted a study to assess the potential of Nintendo Wii in improving the quality of life among older adults in a long-term care facility. Their results indicated that elderly participating in the Wii condition group had a significantly lower level of loneliness than those participating in the other condition group, who played traditional board games. Similarly, another between-group study in the US also reported that playing Wii rather than watching television programs led to a lower level of loneliness. |
| Nnabuko & Anderson (2018)  *The effect of ICT on social support in healthcare: A systematic review*  International Journal on Computer Science and Information Systems | Systematic review (30 papers) | This study aims to review literature on the effectiveness of ICT-based tools and intervention on social support measures with reference to health and illness. | A range in age from 8-83 years. Male & female (n=22), female (n=7), male (n=1) | RCT (n=19), Quasi-experimental studies (n=5), pre- and post-trials (n=2), pilot studies (n=2), feasibility trial (n=1), exploratory online survey (n=1). | Information and Communication Technology (ICT), like Facebook, computer-telephone integrated technology, telehealth technology (e.g., skype, telephone), video technology. | Psychosocial outcomes (self-esteem, empowerment, self-efficacy, depression, quality of life, loneliness). Psychological outcomes (stress, memory & behaviour problems, global cognitive impairment, caregiver burden, symptom distress). Outcomes on the participants’ ability to function (activities of daily living (ADL), information competence, self-care behaviours). Perceptions of intervention (caregiver’s perception of experience, engagement, satisfaction with care). Medical and physical outcomes (blood pressure, medication adherence, asthma control, physical activity, internalized homophobia, body mass index (BMI), Dyspnea assessment, immunologic & viral indicators). | The results show that ICT interventions have a statistically significant positive impact on social support measures. Social network was found to have the most impact on social support measures. 90% of the reviewed studies reported health outcomes similar to the results reported for social support outcomes. Of the included thirty studies, twenty-three (77%) reported a positive impact of ICT intervention on social support measures while seven (23%) reported no impact of ICT intervention on social support. |
| Fuss, Dorstyn, Ward (2019)  *Computer-mediated communication and social support among community-dwelling older adults: A systematic review of cross-sectional data*  Australasian journal on ageing | Systematic review of cross-sectional data (17 studies) | To systematically assess the relationship between computer‐mediated communication and social function in older adults aged 55 or older. | Community dwelling older adults aged 55 or old er (mean age 74.2 (SD 7.1)), range from 55 to 105. Gender; overall 61% male, 39% female | 15 journal articles and 2 dissertations were included in this review. All but 2 studies were cross-sectional, with 1 cohort study and 1 RCT. | Focused on the use of computer-mediated communication (as a primary or secondary outcome) and actual or perceived social functioning. Computer‐mediated communication was broadly defined as the use of digital technologies to facilitate human communication—using binary categorisation (i.e., yes/no use), a numerical rating scale, or in units of time. Any person‐to‐person telephone calling which did not require the use of digital technology (e.g., audio‐only calling) was excluded, given that previous research has suggested that some older adults do not consider telephones (including smartphones) to be a type of digital technology. | Focused on social functioning - defined as any measure of social support or social connectedness. Not direct measure of loneliness - using outcomes such as The Social Provision Scale (SPS), 6-item Lubben Social Network Scale (LSNS-6), Multidimentional Scale of Perceieved Social Support (MSPSS), Social Support Appraisal Scale (SSAS), etc. | The results provide some evidence for a positive, albeit subtle, relationship between these constructs, particularly when computer‐mediated communication is assisted by platforms designed to aid social interaction (e.g., instant messenger and social networking sites). Notably, few studies included a measure of social connectedness, relying primarily on social support as a correlate of computer‐mediated communication. One study assessed loneliness in particular: A single negative relationship was reported among Zhang and Kaufman's 52 sample of online gameplayers in a community‐centred (i.e., social) massively multiplayer online role‐playing game. Higher frequency of gameplay (measured as average number of hours, per day) was associated with higher levels of social loneliness and reduced support from family and friends. In contrast, participants who experienced higher quality gameplay (specifically guild play, where gameplayers work with other gameplayers to progress in the game) identified higher levels of social support (as well as a sense of belonging, and lower levels of loneliness and depression) independent from how much time they spent gaming. |
| Ibarra, Baez, Cernuzzi, Casati (2020)  *A Systematic Review on Technology-Supported Interventions to Improve Old-Age Social Wellbeing: Loneliness, Social Isolation, and Connectedness*  Journal of Healthcare Engineering | Systematic review (25 articles) | Focus on interventions enabling long-distance interactions through technology-mediated communication, targeting loneliness and social isolation in old age. The objective is to identify the findings and limits of the knowledge acquired so far and to emphasize areas where further research is needed. | Participants aged 65 and older, or with a mean participant age above 65 years of age. Gender: n/a | 6 interventions conducted qualitative studies, relying on direct or indirect (e.g., reports by staff) observation, questionnaires, and interviews, 19 interventions conducted quantitative studies, although only five were randomized controlled trials (RCTs) and one was a group randomized trial | Interventions enabling long-distance interactions through technology-mediated communication (e.g., computer and internet training, communication technologies). | Six interventions conducted qualitative studies, relying on direct or indirect (e.g., reports by staff) observation, questionnaires, and interviews. The other 19 interventions conducted quantitative studies, although only five were randomized controlled trials (RCTs) and one was a group randomized trial. Most interventions measured baseline conditions with some form or variant of the UCLA (n =13) or De Jong Gierveld (n = 4) loneliness scales. There were 13 interventions that considered loneliness as a primary outcome, one of which also considered social isolation. Another 12 had loneliness and/or social connectedness as secondary outcomes. | Computer and Internet training was the dominant strategy, allowing access to communication technologies, while in recent years, we see more studies aiming to provide simple, easy-to-use technology. The technology used was mostly off-the-shelf, with fewer solutions tailored to older adults. Social interactions targeted mainly friends and family, and most interventions focused on more than one group of people. |
| Casanova, Zaccaria, Rolandi, Guita (2021)  *The Effect of Information and Communication Technology and Social Networking Site Use on Older People's Well-Being in Relation to Loneliness: Review of Experimental Studies*  Journal of Medical Internet Research | Qualitative review using experimental or quasi-experimental design (11 articles) | The purpose of this review is to contribute to the literature debate on the effect of SNS use on older people’s well-being with specific attention on loneliness, focusing on experimental and quasi-experimental studies | Participants: mean age of 71 years, with 4 studies reporting a mean age over 81 years. Gender: n/a | 9 experimental studies with randomized sampling, 2 quasi-experimental studies, 2 pilot studies, 1 crossover study, 1 integrated the quantitative study with a short qualitative interview at follow-up. | All experimental studies used training classes on PC or SNS (social network services) as the main part of the intervention. Most interventions included the provision of extra incentives to support ICT use (e.g., tutoring and exercise sections - some studies preferred online tutoring, others support and coaching by visiting volunteers). | Loneliness was analysed as a specific well-being outcome. International validated scales were the most used measurement tools. The 44 scales and/or tests were summarized in 5 categories: 1) aspect of social relationship life, 2) neuropsychological conditions, 3) clinical and physical well-being, 4) psychological well-being, 5) ICT attitude and use. Three studies used the UCLA Loneliness Scale as a single perceived social measurement tool, while 2 studies combined that scale with others. | The analysis of the selected articles showed that: (1) ICT use is positively but weakly related to the different measures of older people’s well-being and loneliness, (2) overall, the studies under review lack a sound experimental design, (3) the main limitations of these studies lie in the lack of rigor in the sampling method and in the recruitment strategy. |
| Boulton, E., Kneale, D., Stansfield, C., Heron, P. N., Sutcliffe, K., Hayanga, B., ... & Todd, C. (2021)  *Rapid systematic review of systematic reviews: what befriending, social support and low intensity psychosocial interventions, delivered remotely, may reduce social isolation and loneliness among older adults and how? [version 2].*  F1000Research. | Rapid systematic review of reviews (18 systematic reviews) | This rapid review examines evidence specifically on whether befriending, social support, and low intensity psychosocial interventions delivered remotely can reduce social isolation or loneliness among older adults. | Included adults aged 50+. Results showed a combined age range of 50-95. Gender not specified. | RCTs, quasi-experimental cohort studies, survey studies, and qualitative (semi-structured interviews and focus groups) were all represented. | (i) supported video communication; (ii) online discussion groups and forums; (iii) telephone befriending; (iv) social networking sites; and (v) multi-tool interventions. | There was a range of different outcome measures within the reviews (e.g., UCLA Loneliness scale), although all contained some measure of loneliness or social isolation. | They synthesised evidence from five systematic reviews and 18 primary studies. Remote befriending, social support and low intensity psychosocial interventions took the form of: (i) supported video communication; (ii) online discussion groups and forums; (iii) telephone befriending; (iv) social networking sites; and (v) multi-tool interventions. The majority of studies utilised the first two approaches, and were generally regarded positively by older adults, although with mixed evidence around effectiveness. Focussing on processes and mechanisms, using Intervention Component Analysis (ICA) and Qualitative Comparative Analysis (QCA), they found that the interventions that were most successful in improving social support: (i) enabled participants to speak freely and to form close relationships; (ii) ensured participants have shared experiences/characteristics; (iii) included some form of pastoral guidance. The findings highlight a set of intervention processes that should be incorporated into interventions, although they do not lead us to recommend specific modes of support, due to the heterogeneity of interventions. |
| Choi & Lee (2021)  *Trends and Effectiveness of ICT Interventions for the Elderly to Reduce Loneliness: A Systematic Review*  Healthcare | Systematic review (23 studies) | To investigate the development trends of and summarize the effects of ICT interventions designed for the elderly to reduce social isolation and loneliness. | (Predefined inclusion criteria: (a) Studies involving elderly people aged 60 or older). Results does not replicate exact age of participants - only stating 'elderly people'. Gender: n/a | (Predefined inclusion criteria: Studies including protocols and reviews to consider the latest ICT interventions). Included: 23 studies, including 4 RCTs, 2 non-RCTs, 5 'before studies', 3 mixed-methods, 1 observational and qualitative study, 2 reviews, 2 RCT protocols. | (Predefined inclusion criteria: Studies conducting ICT interventions designed for the elderly to reduce loneliness and social isolation). The interventions ranged from different types listed in table: animal robots (5 studies), humanoid agent (2 studies), mobile robot (4 studies), exercise game (3 studies), interpersonal communication (2 studies), online social platform (7 studies). | Loneliness indicators (UCLA Loneliness Scale, 20 to 80; Ando, Osada, and Kodama Loneliness Scale, 0 to 10; Short Form of UCLA Loneliness Scale; De Jong Gierveld Loneliness Scale, 0 to 6), lower scores of the social isolation tools (Friendship Scale, 0 to 24) represent a decreasing level of loneliness, while higher scores indicate alleviated levels of social isolation. Usability indicators such as usefulness and usability (Technology Acceptance Questionnaire), higher scores and values meant that the ICT interventions were easy to use. Perceptions of ICT interventions and attitudes toward technology (5 to 25), higher scores meant more positive perceptions. Social cognition (Young Schema Questionnaire) and perceived vulnerability (Perceived Vulnerability Scale, 1 to 6) were examined, and lower scores of the indicators indicated more positive attitude toward ICT interventions. | The principal finding of this systematic review is that the trend of development is changing from animal robots to online social platforms and from simple emotional support to a multifaceted system that promotes social participation, cognition, physical activity, nutrition, and sleep. Secondly, the results showed that ICT interventions for the elderly are being developed to alleviate loneliness among the elderly and increase social participation. Thirdly, the systematic review revealed that the elderly increased their use of ICT interventions and have a positive attitude towards it. |
| Gasteiger, Loveys, Law, Broadbent (2021)  *Friends from the Future: A Scoping Review of Research into Robots and Computer Agents to Combat Loneliness in Older People*  Clinical Interventions in Aging | Scoping review (29 studies) | This scoping review aimed to synthesize and report evidence on the effectiveness of interventions using social robots or computer agents to reduce loneliness in older adults and to explore intervention strategies. | Aged between 62 and 85.8 years. Women participated more than men; female participants ranged from 50% to 100%. | Observational studies (18), experimental studies (11). | 24 studies investigated the effect of social robots (most commonly used: Paro, AIBO, MARIO, iRobi), five studies used virtual agents (Care Coach, Tanya, AlwaysOn System). | Loneliness measured through both qualitative and quantitative data. The latter included UCLA Loneliness Scale, the modified Lexington Attachment to Pets Scale, the Multidimensional Perceived Social Support Scale, the Ando-Osada-Kodama (AOK) Loneliness Scale, the Norbeck Social Support Questionnaire, and the Relationship Closeness Inventory. | The majority of results showed that robots or computer agents positively impacted at least one loneliness outcome measure. Some unintended negative consequences on social outcomes were reported, such as sadness when the robot was removed. Overall, the interventions helped to combat loneliness by acting as a direct companion (69%), a catalyst for social interaction (41%), facilitating remote communication with others (10%) and reminding users of upcoming social engagements (3%). |
| Gorenko, Moran, Flynn, Dobson, Konnert (2021)  *Social Isolation and Psychological Distress Among Older Adults Related to COVID-19: A Narrative Review of Remotely Delivered Interventions and Recommendations*  Journal of Applied Gerontology | Narrative review (allows an integrated evaluation of interventions, and examination of the appropriateness of existing interventions) (19 studies). | To provide a summary of the literature on remotely-delivered interventions for older adults that target loneliness and/or psychological distress (e.g., depression, anxiety), with three primary aims as follows: (a) identify remotely-delivered evidence-based interventions for social isolation and psychological distress that are efficacious and feasible for delivery to older adults during a pandemic, (b) provide a comprehensive overview of such interventions appropriate for implementation by individuals with differing education and training (e.g., family members, community volunteers, mental health professionals), and (c) identify barriers to remote-delivery of reviewed interventions for older adults and provide recommendations that acknowledge unique considerations among the aging population and the COVID-19 pandemic. | Not specifically stated - only 'older adults' (no overview of age or any other characteristics of participants). From table: age span from 75/79/50+/57 to 85/69 to 85/ 65+/65-89/55+/60+/45+/. Gender: n/a | RCTs (several), qualitative interviews, pilot projects, case study, randomized crossover study, non-RCT pilot study, RCT pilot studies. | Focus on remotely delivered interventions for older adults that target loneliness and psychological symptoms. Ranging from family interaction through video calls, community befriending interventions, interest-based education programs, internet and social media use training, self-guided interventions (such as Internet-based cognitive behavioural therapy), different types of clinician-led/guided interventions by telephone, video call, Internet. | A range of outcomes was measured in studies reviewed (e.g., loneliness, depression), and outcome evaluations included qualitative interviews and validated outcome measures (e.g., Geriatric Depression Scale [GDS]). No studies evaluated internet use as a stand-alone intervention (without a training component). | Results showed that in cross-sectional research, direct communication via social networking websites (e.g., exchanging direct messages) was associated with reduced loneliness, while passive engagement was associated with greater loneliness. Older adults also reported the importance of traditional communication channels (i.e., telephone), suggesting that social media use augments but may not replace traditional modes of communication. |
| Heins, Boots, Koh, Neven, Verhey, de Vugt (2021)  *The effects of technological interventions on social participation of community‐dwelling older adults with and without dementia: A systematic review*  Journal of Clinical Medicine | Systematic review (37 reports, covering 36 studies) | The present systematic review aims to provide a comprehensive overview of the effects of technological interventions that address social participation in community-dwelling older adults with dementia. | Aimed at community-dwelling older adults (defined as aged 55 and older) with or without cognitive impairment. Sample sizes (M = 53.86, SD = 72.23) ranged from 5 to 300, with a majority of the participants being female. | Qualitative study design (n=14), quantitative study design (n=12), mixed-methods studies (10). | Studies were heterogenous in terms of intervention characteristics. Most studies evaluated social networking technology and ICT training programs (details: A third of the included studies (n = 12) focused on communication and social networking technology and 10 evaluated ICT training programs). Only three studies focused on people with dementia. Few studies addressed mobile applications (n = 4) and gaming technology (n = 4). The remaining studies examined the effect of activity-based musical engagement with iPads, a tablet based language training program, the provision of Internet access, technology assisted self-monitoring of physical activity, a Personal Reminder Information and Social Management (PRISM) system, and telecare. | Only 2 of the 36 included studies highlighted an explicit primary intervention aim to increase social participation. Numerous studies mentioned addressing other social outcomes, such as social isolation and loneliness (n=12). Loneliness was the most frequently measured psychosocial outcome identified in quantitative and mixed methods studies, followed by perceived social support and social isolation. Interestingly, the variables of loneliness, social isolation, and (perceived) social support were not measured coherently. | Quantitative findings showed limited effects on loneliness, social isolation, and social support. Nevertheless, several benefits related to social participation were reported qualitatively. Social interaction, face-to-face contact, and intergenerational engagement were suggested to be successful elements of technological interventions in improving the social participation of community-dwelling older adults. |
| Jin, Liu, Bai, Bai (2021)  *The effectiveness of technology-based interventions for reducing loneliness in older adults: A systematic review and meta-analysis of randomized controlled trials*  Frontiers in Psychology | Systematic review (6 papers) | The present study aimed to conduct a systematic review of existing studies that examined the effectiveness of technology-based interventions for reducing loneliness in older adults. | Participants 60 and over, 64-75, 70-93. Gender: n/a | RCTs. | Smartphone, videoconference interaction, computer operation and internet use, teleconferences | UCLA Loneliness Scale (n=4), Loneliness questionnaire (n=1), the De Jong Gierveld Loneliness Scale (n=1) | A total of 391 participants from six RCTs were included in the review. Of these, three studies were rated as low-quality, and the remaining three were rated as moderate-quality studies. The meta-analysis showed that the evidence regarding the effects on loneliness of technology-based interventions compared with control groups was uncertain and suggested that technology-based interventions resulted in little to no difference in loneliness reduction compared to control groups. Two types of technology-based interventions were identified: smartphone-based video calls and computer-based training with Internet usage. The subgroup analysis found low-quality evidence to support the effectiveness of both intervention types. |
| Latikka, Rubio-Hernández, Lohan, Rantala, Fernandez, Alitinen, Oksanen (2021)  *Older adults` loneliness, social isolation, and physical information and communication technology in the era of ambient assisted living: A systematic literature review*  Journal of medical internet research | Systematic literature review (23 papers) | The aim is to gain insight into how technology can help overcome loneliness and social isolation other than by fostering social communication with people and what the main open-ended challenges according to the reviewed studies are. | In some articles is age not applicable (n=1) or multiple (n=4). In some studies, the mean age is over 80 years (n=5), but the range variates in these studies from 65 – 98 years. Other studies have a mean/median age around 70 (n= 11) but the range variates from 62 – 81 years. Gender not specified. | Quantitative (n=13), qualitative (n=3), mixed methods (n=7) | The 2 main areas of the reviewed research comprised “detection and prediction” and “alleviation” of older adults’ loneliness and social isolation using physical ICT. | UCLA Loneliness Scale (n=12), Dong Jong Gierveld Loneliness Scale (n=4), Lubben Social Network Scale (n=2) | ICT solutions such as smart homes can help detect and predict loneliness and social isolation, and technologies such as robotic pets and some other social robots can help alleviate loneliness to some extent. The main open-ended challenges across studies relate to the need for more robust study samples and study designs. Further, the reviewed studies report technology- and topic-specific open-ended challenges. |
| Newman, Stoner, Spector (2021)  *Social networking sites and the experience of older adult users: a systematic review*  Ageing & Society | Systematic review (21 papers) | The aim of the review was to identify, characterise and summarise existing research on SNS use from an older adult perspective. | A range from 53 – 103 years. Over 50 % women (n=15), more than 50 % men (n=3), not reported (n=2), Unclear (n=1) | The majority of studies used correlational or descriptive methods, and two used an experimental design. Of the descriptive and correlational studies, one study used a longitudinal design. Six studies employed qualitative methods, and one study used mixed methods. | The use of social networking sites (SNSs) from an older adult perspective (e.g., Facebook, Twitter, MySpace) | Aspects of social relationships that have relevance to psychological wellbeing (n=11). Three studies examined the relationship between SNS use and cognitive function (n=3). | Papers suggest that SNSs are used by older adults to maintain connections to people they are already close to rather than being used as a vehicle to form new ties. Since most papers were of low or medium quality, these findings suggest that the relationship between SNS use and wellbeing amongst older adults is currently inconclusive. The evidence for the relationship between SNS use and cognitive function based on this very small number of studies was therefore mixed. There was some preliminary indication that learning how to use an SNS site had benefits for an aspect of executive function. |
| Shah, Nogueras, van Woerden, Kiparoglou (2021)  *Evaluation of the Effectiveness of Digital Technology Interventions to Reduce Loneliness in Older Adults: Systematic Review and Meta-analysis*  Journal of Medical Internet Research | Systematic review (6 studies) | The objective of this study is to assess the effectiveness of digital technology interventions (DTIs) in reducing loneliness in older adults. | Average age: 71 - 78 years. Studies varied in the proportion of male and female participants (female: mean 66%, SD 16%; range 46%-81%; male: mean 25%, SD 9%; range 19%-42%). | 1 before and after study and 5 clinical trials (4 randomized clinical trials and 1 quasi-experimental study) | Digital technology interventions (DTIs) | Loneliness | The overall effect estimates showed no statistically significant difference in the effectiveness of DTIs compared with that of usual care or non-DTIs at follow-up at 3 months (SMD 0.02; 95% CI −0.36 to 0.40; P=.92), 4 months (SMD −1.11; 95% CI −2.60 to 0.38; P=.14), and 6 months (SMD −0.11; 95% CI −0.54 to 0.32; P=.61). The quality of evidence was very low to moderate in these trials. |
| Wister, O'Dea, MA, Cosco (2021)  *Technological interventions to reduce loneliness and social isolation among community-living older adults: A scoping review.*  Gerontechnology | Scoping review (26 studies) | The primary objective of this study is to provide a comprehensive overview of the types of technological interventions that have been developed to reduce loneliness and/or social isolation for community-dwelling older adults. The secondary objective is to compare via mapping the technological intervention article characteristics in terms of date of publication, country of publication, study design, sample characteristics, loneliness/ social isolation measure, and efficacy/support for the intervention. | This scoping review was comprised of studies with participants aged 60 or older, or with a mean age of 65 and above. Twenty-three included studies had study populations that were over 50% female, two included populations that were >50% male, and one study did not report on sex. | The majority (16 out of 26) of studies were either quasi experimental designs or descriptive (i.e., post-test only, pre-post-test design with no control group, non-equivocal control group, time-series design, or were descriptive. The remaining 10 studies employed a randomized clinical trial design. | Identified 26 technological approaches to reducing loneliness and social isolation that met our criteria. They found three groupings; (1) computer and tablet-based competence training; (2) health-oriented technical interventions; and 3) Video games and animatronic pets' interventions. | The majority of the studies included in this scoping review (20 out of 26) measured changes in loneliness outcomes among older adult participants (e.g., different versions of the UCLA loneliness scale, Changes in social isolation outcomes among participants were measured using instruments including the 4-item Social Interaction Subscale of the Duke Social Support Index (DSSI-I), and the Patient Reported Outcome Measurement Information System (PROMIS)- Social Isolation (6-item). Open ended and semi-structured interviews, as well as self-report questionnaires, were also utilized to measure loneliness and/or social isolation in a small number of studies. | The majority of technological interventions (15 out of 26) were effective in demonstrating support for reductions in loneliness and/or social isolation among older adults. Issues of accessibility, technology literacy, and complexity of the intervention were found to act as barriers to uptake. |
| Balki, E., Hayes, N., & Holland, C. (2022).  *Effectiveness of technology interventions in addressing social isolation, connectedness, and loneliness in older adults: systematic umbrella review*.  JMIR aging | Systematic umbrella review (24 publications) | This study aimed to identify, synthesize, and critically appraise the effectiveness of technology interventions improving social connectedness in older adults by assessing the quality of reviews, common observations, and derivable themes. | Included adults aged ≥50 years in community and residential settings. Gender not specified. | The 21 selected reviews included 16 (76%) systematic reviews, 2 (10%) integrative reviews, 2 (10%) scoping reviews, and 2 (10%) meta-analyses. | The included reviews were dedicated to information and communications technology (ICT; 11/24, 46%), videoconferencing (4/24, 17%), computer or internet training (3/24, 12%), telecare (2/24, 8%), social networking sites (2/24, 8%), and robotics (2/27, 8%). | When analysing these quantitative primary studies, the reviews commonly applied validated tools, such as the University of California Los Angeles (UCLA) Loneliness Scale (or a modified version) and the De Jong Gierveld Scale. The UCLA was the most tested dependent variable. Among various other measures were the Social Support Scale by Schuster and Hunter, Social and Emotional Loneliness Scale, and Multidimensional Scale of Perceived Social Support. Social connectedness was sometimes measured using the holistic Social Connectedness Scale by Lee and Robin, which is regarded as a comparatively reliable measure. | Although technology was found to improve social connectedness, its effectiveness depended on study design and is improved by shorter durations, longer training times, and the facilitation of existing relationships. ICT and videoconferencing showed the best results, followed by computer training. Social networking sites achieved mixed results. Robotics and augmented reality showed promising results but lacked sufficient data for informed conclusions. The overall quality of the studies based on GRADE was medium low to very low. Technology interventions can improve social connectedness in older adults. The specific effectiveness rates favour ICT and videoconferencing, but with limited evidence, as indicated by low GRADE ratings. |
| Döring, Conde, Brandenburg, Broll, Gross, Werner, Raake (2022)  *Can Communication Technologies Reduce Loneliness and Social Isolation in Older People? A Scoping Review of Reviews*  International Journal of Environmental Research and Public Health | Scoping review of reviews (28 research reviews) | This scoping review of reviews aims to summarize the communication technologies (CTs) (review question RQ1), theoretical frameworks (RQ2), study designs (RQ3), and positive effects of technology use (RQ4) present in the research field. | Included participants 55 years of age or older without cognitive impairment. Gender n/a. | Of the 28 reviews, 26 (93%) covered primary studies with quantitative-experimental designs. | Internet and computer were the most researched communication technologies (23 reviews or 82% each), followed by videoconference systems such as Zoom or Skype (16 reviews, 57%), email (13 reviews, 46%), telephone (12 reviews, 43%), and social robot (10 reviews, 36%). Augmented reality (AR) or virtual reality (VR) systems were covered by only one of the 28 included reviews (4%). | Outcome measures of qualitative primary studies entailed, for example, qualitative assessments of the perceived benefits of general internet use to overcome loneliness and/or social isolation, perceptions of social presence when using CTs, reports of reconnecting with family through off-the-shelf applications, and factors that impact the easy adoption of digital devices and software among older adults. Outcome measures of quantitative-observational primary studies included associating technology use with level of loneliness and/or social isolation or comparing loneliness and/or social isolation levels between groups of users and non-users of technology. Outcome measures for quantitative-experimental designs were changes in loneliness and/or social isolation levels after the interventions. | The majority of the included reviews addressed general internet and computer use (82% each) (RQ1). Of the 28 reviews, only one (4%) worked with a theoretical framework (RQ2) and 26 (93%) covered primary studies with quantitative-experimental designs (RQ3). The positive effects of technology use were shown in 55% of the outcome measures for loneliness and 44% of the outcome measures for social isolation (RQ4). While research reviews show that ICTs can reduce loneliness and social isolation in older people, causal evidence is limited and insights on innovative technologies such as augmented reality systems are scarce. |
| Fu, Z., Yan, M., & Meng, C. (2022).  *The effectiveness of remote delivered intervention for loneliness reduction in older adults: A systematic review and meta-analysis.*  Frontiers in Psychology | Systematic review and meta-analysis (13 studies) | This study aimed to conduct an updated meta-analysis and systematic review on the loneliness reduction obtained by remotely delivered intervention for loneliness in older adults. | Included older adults who are over the age of 65 years | Randomized controlled trials (n=13). | Six studies used telephone call intervention, three studies used video call intervention, four studies used the computer or internet-based intervention. | The loneliness measurement tools used were the UCLA loneliness scale, the De Jong-Gierveld loneliness scale, and PROMIS-L. | This systematic review and meta-analysis demonstrate that remotely delivered intervention can result in loneliness reduction. The subgroup analysis suggested remotely delivered intervention had a superior effect on loneliness when delivered from an individual, by video call, using increasing social support or maladaptive social cognition treatment strategy, to older adults under LTC or social isolation circumstances, with measurement time points below 6 months, when compared with different control groups. These favourable effects of remotely delivered intervention involve complex interactions with the patient, including empathy, intention, care, and attention, that cannot be achieved by medications alone or by no intervention. |
| Chen, Wood, Ysseldyk (2022)  *Online Social Networking and Mental Health among Older Adults: A Scoping Review*  Canadian Journal on aging-revue Canadienne du vieillissement | Scoping review (52 articles) | The objective of this scoping review was to gather, summarize, and better understand the existing literature on the use of online social networking and associations with mental health among older adults, in order to potentially inform future interventions, programming, and policies. | Minimum sample age: 50 to 75, sample sizes ranged from 8 to 7839. Gender: N/A | The majority of studies used a quantitative (n = 28), qualitative (n = 8), or mixed methods approach (n = 8). The remaining articles identified in this scoping review were systematic and/or literature reviews (n = 8). | (Predefined inclusion criteria: interventions that focus on the link between aging, mental health (e.g., social isolation, loneliness, depression) and social technology (e.g., e-mail. social technology, social media). Excluded research examining technology that did not fit with social networking factors (e.g., assistive living technologies, robotics)). Included papers described the following interventions: SNS (social networking site) usage / online social networking, ICT usage. | Research methods to evaluate the use of online social networking, levels of loneliness and depression, overall feelings of social connectedness, and well-being included self-report scales and interviews. | Five common themes were identified: (1) enhanced communication with family and friends, (2) greater independence and self-efficacy, (3) creation of online communities, (4) positive associations with well-being and life satisfaction, and (5) decreased depressive symptoms. |
| Kusumota, Diniz, Ribeiro, da Silva, Figueira, Rodrigues, Rodrigues (2022)  *Impact of digital social media on the perception of loneliness and social isolation in older adults*  Revista Latino-Americana de Enfermagem | Integrative review (IR), (11 articles) | The present study aimed to synthesize knowledge about the use of social media and the perception of loneliness and/or social isolation in older adults. | No specific information about age of participants in included papers. | Quantitative (n=5), mixed methods (n=3) and qualitative (n=3) designs. | Use of social media: it was possible to identify different types of technologies used in the studies. For a better understanding, these technologies were divided into three categories, namely: “Internet use”, encompassing social networking sites, internet and applications, found in four (4) studies; “communication devices”, which covered information regarding the use of smartphones, tablets and iPads, with four (4) studies included in this category; and “types of communication” which covered the means of interpersonal communication in the digital age, such as video calls, emails and computer programs, with three (3) studies in this category. | Quantitative studies: different questionnaires, e.g., 1) Loneliness Scale and Mental Health Inventory, 2) Lubben Social Network Scale (LBNS6), De Jong Gierveld Loneliness Scale (DJG6), Warwick-Edinburgh Short Mental Well-Being Scale (SWEMWBS), Life Satisfaction Scale, Independence, using a question from the index of the measure Investigating Choice Experiments for the Preferences of Older People Capability measure (ICECAP) and questions from the Personal eHealth Readiness Questionnaire (PERQ). 3) Friendship Scale, Loneliness Scale, Interpersonal Support Evaluation List, Lubben Social Network Index, Quality of Life Scale, Perceived Vulnerability Scale, and the SF-36. 4) Likert-type scale was used, with questions taken from the Loneliness Scale (UCLA). 5) Social Support Scale (Duke Social Support Scale) and Solitude Scale (UCLA) were performed. 6) Likert type scale. Loneliness was assessed using the Revised Loneliness Scale (R-UCLA), psychological stress using the Kessler Scale, and sense of community using the Brief Sense of Community Scale (BSCS). 7) Loneliness Scale (UCLA), Geriatric Depression Scale, Quality of Life (SF-36). Qualitative studies: e.g., 1) multiple-choice questions and open-ended questions. 2) Ethnographic approach consisting of observations, unstructured interviews, writing of memos, feedback forms and reflective diaries was undertaken to collect. 3) Discussion in a focus group. Content analysis was performed to evaluate the group discussions and social media data. 4) semi structured interviews with participants and family members. | There were positive results (63.6%) regarding the use of social media to minimize the perception of loneliness and/ or social isolation in the older adults. Seven studies (63.6%) included showed positive results in the use of social media to minimize the perception of loneliness and/or social isolation of the older adults. The studies showed that a simplified approach and the prior training of the older adults in the use of information and communication technology (ICT) had positive results in terms of interaction with their family members, improved quality of life, access to information and greater social participation. Therefore, the results demonstrate that the use of social media has an impact on reducing the perception of loneliness and/or social isolation in older adults. Conversely, 36.3% (n=4) of the studies highlighted the need to develop more robust studies to address the impact of low self-esteem in the older adult, and the low participation of the family members and social support, as barriers to the use of technologies and the internet. |
| Rodrigues, Han, Su, Klainin-Yobas, Wu (2022)  *Psychological impacts and online interventions of social isolation amongst older adults during COVID-19 pandemic: A scoping review*  Journal of Advanced Nursing | Scoping review (33 articles) | To summarise the psychological impacts of social isolation amongst older adults during COVID-19 and review the benefits and limitations of online interventions used to combat social isolation. | Inclusion criteria aged 55 years old and above. Gender specified only in few included articles. | Case study (n= 3), Phenomenology (n=2), Descriptive qualitative (n=1), Narrative review (n=2), Cross sectional (n=8), Quasi-experimental (n=1), RCT (n= 1), Prevalence= (n=2), Discussion paper (n=5), Review paper (n=2), Commentary (n=6). | Online interventions (different telehealth solutions for medical services, online home-based physical activity programmes, e.g., apps, TV-assistDem, remotely delivered psychological interventions, other online interventions, telephone outreach) | Different outcomes (e.g., UCLA loneliness scale, perceived burdensome, etc.) | Four themes and eight sub-themes emerged: (1) negative impacts and experiences of older adults during social isolation, (2) adopting coping behaviours in the midst of COVID-19, (3) online interventions to combat the consequences of social isolation, (4) barriers to online intervention. Online interventions, which could be a new normal in the COVID era, were beneficial in combating social isolation. Strategies by various stakeholders were recommended to tackle the barriers of online interventions. |
| Sen, Prybutok, Prybutok (2022)  *The use of digital technology for social wellbeing reduces social isolation in older adults: A systematic review*  SSM - Population Health | Systematic review (narrative analysis to identify themes) (25 studies). | The purpose of this systematic review is to evaluate the extent to which technology can be used to address the problem of social isolation (SI) and enhance the quality of life for older adults. | Age and gender not specified. | Interview-based qualitative evaluation (n=10), Surveys of older adults 'use of technology (n=4), Mixed-methods evaluation (n=1), quantitative evaluation (n=10) | Focused on research into older adults' use of digital networks (i.e., older adults' satisfaction with the use of technology, technologies related to wellbeing of older adults, frequency and history of internet use by seniors, benefits of the use of technology, accessibility of technology to older adults, technology training and the conditions for use based on the perceived needs of the users). Software incorporated social interaction features (e.g., photographs, videoconferences with family members and friends). | Social isolation, social participation, but not mentioned specifically instruments (14 out of 25 reviewed articles did not provide clear or measurable definitions of social interaction or SI, which adds to difficulty in developing measurable interventions in research to address SI in the future). | Engagement of older adults at the community-level, following best practices from the Community-Based Participatory Research can facilitate effective practices to deliver technology based social isolation interventions and increase digital use self-efficacy in older adults. Mobile technology-based applications not only help families to stay connected, but also link older adults to resources in healthcare and encourage physical and mental well-being. Use of technology devices address cognitive, visual, and hearing needs, and increase digital use self-efficacy in older adults, particularly helpful during necessary social distancing or self-quarantine during the COVID-19 pandemic. |
| Thangavel, Memedi, Hedström (2022)  *Customized Information and Communication Technology for Reducing Social Isolation and Loneliness Among Older Adults: Scoping Review*  JMIR Mental Health | Scoping review (39 studies) | The aim of this review is to explore ICT solutions for reducing social isolation or loneliness among older adults, the purpose of ICT solutions, and the evaluation focus of these solutions. | Most of the studies (16/39, 41%) included older adults with a starting age of 65 years, followed by ≥60 years (8/39, 21%), ≥55 years (4/39, 10%), ≥70 years (3/39, 8%), ≥75 years (2/39, 5%), and ≥50 years (2/39, 5%), and the remaining studies did not mention the starting age of their study population. Gender not specified. | Not mentioned | Information and communication technology (ICT): They found 7 different types of ICT solutions. These were: social networks were mainly proposed by many studies (14/39, 36%), followed by video chat (8/39, 21%), messaging services (8/39, 21%), robotics (6/39, 15%), virtual spaces or classrooms with messaging capabilities (7/39, 18%), games (4/39, 10%), and content creation and management (2/39, 5%). | Loneliness, social isolation: Loneliness was measured using the University of California, Los Angeles Loneliness Scale in 45% (5/11) of the studies that measured loneliness, and 18% (2/11) used the revised version of the same scale. Jansen et al used the De Jong Gierveld and Kamphuis 11-item loneliness scale, whereas Brandenburgh et al used the short version of the same scale that comprises 6 items. Morganti et al used the Italian Loneliness Scale, which has 18 items that are grouped into three subscales: emotional loneliness, social loneliness, and general loneliness. | The mapping of purposes of ICT solutions with problems found among older adults indicates that increasing social communication and social participation can help reduce social isolation problems, whereas fulfilling emotional relationships and feeling valued can reduce feelings of loneliness. In terms of customized ICT solution types, we found the following seven different categories: social network, messaging services, video chat, virtual spaces or classrooms with messaging capabilities, robotics, games, and content creation and management. Most of the included studies (30/39, 77%) evaluated the usability and acceptance aspects, and few studies (11/39, 28%) focused on loneliness or social isolation outcomes. |
| Todd, Bronwyn, Allyson (2022)  *Using information and communication technology learnings to alleviate social isolation for older people during periods of mandated isolation: A review*  Australasian Journal on Ageing | A review (15 studies) | To examine the effectiveness of information and communication technologies (ICTs) in reducing social isolation in older people and draw recommendations from previous literature appropriate for informing ICT use in future mandated periods of isolation. | Average between 66 - 82 years. Gender not specified. | RCT (n=2), controlled quasi-experimental study (n=4), qualitative research (n=2), mixed methods (n=6) | ICT intervention (e.g., internet-based applications for phones and tablets, videoconferencing, telephone support networks). | UCLA loneliness scale, DeJong Gierveld, Loneliness Scale and QoL scale | Most studies reported positive impacts on social isolation, but this was identified more in self-reporting compared to changes in baseline measures. The types of ICT used included videoconferencing, Internet-based applications, and purpose-designed applications. A number of factors were also identified throughout the studies that impacted uptake that should be considered when implementing ICT. Overall, the studies included in this review found that most ICT interventions had some impact on the social isolation experienced by older people; however, as previous reviews have found the heterogeneity of interventions (e.g., from videoconferencing robots to telephones), and variety of outcome measures, limits strong conclusions being drawn about the effectiveness of different interventions. |
| Wiwatkunupakarn, Pateekhum, Aramrat, Jirapornchaoren, Pinyopornpanish & Angkurawaranon (2022)  *Social networking site usage: A systematic review of its relationship with social isolation, loneliness, and depression among older adults*  Aging and Mental Health | Systematic review (15 articles) | This review aimed to summarize the relationship between SNS (social networking sites) usage in daily-life routine and depression among older adults aged 60 years and older. Also, to examine the relationship between SNS use and loneliness as well as social isolation as they are considered predisposing factors for depression. | Older adults over 60 years and older. The mean age of the sample varied between 61,5 – 80,7 years. Gender not specified. | Ten observational and five experimental studies. | Use of social networking sites (SNS) (i.e., a subset of social media that helps people to interact with other people online, like Facebook, Twitter, LinkedIn etc). | The measurements of depression, loneliness and social isolation were all subjective. Standard tools such as The Center for epidemiology Studies Depression Scale (CeS-D) (n=4), Patient Health Questionnaire 2 (PHQ-2) (n=2), Beck’s Depression inventory (BDI) (n=1), The Mental Health inventory-5 (MHi-5) (n=1), The University of California, Los Angeles (UCLA) Loneliness Scale (n=1), and others (n=6). | There was a pattern among five observational studies examining SNS usage and loneliness, such that SNS usage was inversely associated with loneliness. However, only three out of five observational studies were able to achieve a statistically significant difference. Three RCTs and one pre-post intervention study examining loneliness did not demonstrate a consistent association between SNS and lower loneliness scores, only one RCT was able to achieve significant results. For social isolation, there were only two observational studies that examined this outcome. There was insufficient evidence to suggest that SNS usage was associated with a lower level of social isolation. |
| Petersen, B., Khalili-Mahani, N., Murphy, C., Sawchuk, K., Phillips, N., Li, K. Z., & Hebblethwaite, S. (2023).  *The association between information and communication technologies, loneliness and social connectedness: A scoping review*  Frontiers in Psychology | Scoping review (54 studies) | The aim of this scoping review was to examine the research that explores how ICTs may be implicated in mitigating loneliness and increasing social connectedness among older adults. | Participant(s) were required to be 60 years of age or older in good general health. Percentage of female ranged from 18 to 100% in each study. | Cross-sectional surveys and interviews/focus groups, quasi-experimental designs, randomized controlled trials, case series, and cohort studies. | Interventions are defined as studies where older adults were interacting or training with an ICT device either alone or in groups over a designated period of time, followed by providing feedback on how the interactions or training impacted their wellbeing via questionnaires or interviews. | Range of different social outcomes including social connectedness, loneliness, social support, social engagement, inclusion, and leisure. | After the examination of 54 articles, they identified three major themes within the literature: (1) ICTs were associated with a reduction in loneliness and increase in wellbeing. (2) ICTs promoted social connectedness by facilitating conversations. (3) Factors such as training, self-efficacy, self-esteem, autonomy, and the design/features, or affordances, of ICTs contribute toward the associations between ICT use and wellbeing. The heterogeneity of methodologies, statistical reporting, the small sample sizes of interventional and observational studies, and the diversity of the experimental contexts underline the challenges of quantitative research in this field and highlights the necessity of tailoring ICT interventions to the needs and contexts of the older users. |
| Zhou, Cheng, Sabran & Zahari (2023).  *User interfaces for older adults to support social interaction through digital technology: a systematic review update.*  Disability and Rehabilitation: Assistive Technology | Systematic review (29 studies) | To update the results related to user interfaces and digital technologies that support the social interactions of older adults. The main research questions are as follows: RQ1: which social interactions in older adults are supported by the identified user interface? RQ2: what digital technologies are used in user interfaces designed for older adults to support social interaction? RQ3: what aspects of user interfaces affect the social interactions of older adults? | The individual or average age of the study population was 60 years and older. Number of females in each study ranged from 2 (sample size=4) to 54 (sample size=102). | 12 of the studies used quantitative methods, 14 used mixed methods, 3 were qualitative. | More than half of the studies were focused on mobile technology (n=15), followed by web-based technologies (n=3), multimodal interactions (n=5), gesture-based interactions (n=2), speech recognition and natural language processing (n=2) and tangible user interfaces (n=2). | The key outcomes of the studies were related to user interface support and influencing social interactions among older adults. | The findings were reviewed in three areas: social interaction of older adults supported by user interface (RQ1), the digital technologies used in the user interface (RQ2), and the effects of user interfaces on the social interactions of older adults (RQ3). Several studies measured the change in loneliness levels of older users after using a user interface. Other studies obtained changes in older adults’ social interactions after using user interfaces through semi-structured interviews, questionnaires, pre-tests and post-tests, observations, and surveys. Results identified four key factors influencing older adults' social interaction experience though the interface, categorized as perceived usefulness, ease of use, accessibility and user preferences and behavior. |
| Lei, Matovic, Leung, Viju, and Wuthrich (2024).  *The relationship between social media use and psychosocial outcomes in older adults: A systematic review.*  International Psychogeriatrics | Review (64 papers in total, whereas 26 studies investigated the relationship between social media use and loneliness, and four studies investigated the relationship between social media use and older adults' social connectedness or relatedness). | 1) To explore the most recent literature (from 2007 to the current date) to examine the impact of older adults’ social media use on a broad range of psychosocial outcomes. 2) To synthesize the effects of social media use on older adults’ psychosocial outcomes across loneliness, depression, anxiety, social connectedness, social isolation, life satisfaction, quality of life, and wellbeing without restriction on participants’ living arrangements or study design. 3) To synthesize the findings regarding the mediators of the relationship between social medial use and older adults’ psychosocial outcomes so that the important components of social media use could be better understood. | Older adults were defined as aged 65 years or older. Percentages of females in each study ranged from 42% to 100%. | Among studies assessing loneliness (n=26); 17 cross-sectional studies, 2 longitudinal studies, 7 interventional studies. Among studies assessing social connectedness and relatedness (n=4); 2 cross-sectional studies, 1 interventional study, 1 longitudinal study. | In this review, social media formats encompassing a wide range of sites, platforms, and apps enable communication through varied formats including sending and receiving text messages, photos, voices, videos, making voice/video calls, video conferencing and creating, sharing and responding to posts through smartphones, tablets, or computers. | Most studies measured loneliness with various forms of the De Jong-Gierveld Loneliness Scale and the UCLA Loneliness Scale. The Social and Emotional Loneliness Scale for Adults. and a single question (n = 2) were also used to measure loneliness. Social connectedness or relatedness was measured with the Social Connectedness Scale, the Balanced Measure of Psychological Needs Scale and a customized scale. | In general, the cross-sectional studies found that greater social media use was associated with lower rates of loneliness (n = 13). This was replicated across a wide range of social media use measures such as user status, frequency of use, number of online applications used, and duration of use. Results of longitudinal studies were mixed, with one of the two studies reporting that more frequent social media use predicted reduced loneliness over time, while the other found no association between time spent on social media and loneliness over time. Similarly, the interventional studies reported mixed results. Only one cross-sectional study found that more frequent social media use was associated with higher relatedness, and the other three studies did not find an association between social media use and social connectedness. |
| Shekelle, Miake‑Lye, Begashaw, Booth, Myers, Lowery, and Shrank (2024)  *Interventions to Reduce Loneliness in Community‑Living Older Adults: A Systematic Review and Meta‑analysis.*  Journal of general internal medicine | Systematic review and meta-analysis (60 studies in total, whereas 10 studies investigated internet training and 7 studies internet-delivered interventions). | The goal was to conduct a systematic review and meta-analysis of diverse interventions to reduce loneliness in a more homogeneous population, namely older community-living adults, with loneliness being measured with a validated measure. | All studies were either restricted to adults that were at least aged 50 or older or had a mean age that was over age 50. Gender not specified. | Technology-based studies (n=17): 12 RCTs, 5 observational studies. | Internet training (consisted of basic computer skills, internet use, email competency, social media, photographs, and video chat applications); internet-delivered interventions (assessed internet-delivered cognitive behavioral therapy or other interventions). | UCLA loneliness scale and De Jong Gierveld loneliness scale | Five RCTs and 5 observational studies provided moderate certainty evidence that internet training was associated with reduced loneliness (standardized mean difference for RCTs = − 0.22, 95% CI− 0.30, − 0.14). Evidence was insufficient to reach conclusions about internet-delivered interventions. |
